# Atomic Cu^2+^ Clusters vs Single Cu^2+^ Atoms as Optimal Cocatalysts on NaTaO_3_ for Enhanced Noble-Metal-Free H_2_O/H_2_ Photocatalysis

**DOI:** 10.1021/acscatal.5c03183

**Published:** 2025-07-23

**Authors:** Anastasia V. Spyrou, Areti Zindrou, Christos Sidiropoulos, Yiannis Deligiannakis

**Affiliations:** Laboratory of Physical Chemistry of Materials & Environment, Department of Physics, 37796 University of Ioannina, Ioannina 45110, Greece

**Keywords:** flame spray pyrolysis, NaTaO_3_, single-atom catalysis, Cu cocatalyst, EPR detection of Cu monomers/clusters, photocatalysis

## Abstract

Engineering of single-atom catalysts (SACs) or subnanoclusters (SnCs) as cocatalysts is a powerful strategy for enhancing photocatalytic performance. However, a more concrete understanding of the role of SAC vs SnC remains a challenge. A prerequisite to achieving this task is a systematic selective synthesis of SACs and SnCs as cocatalysts for a given process. Herein, we have employed a modified flame spray pyrolysis (FSP) process for deposition of either single Cu^2+^ atoms or Cu^2+^ subnanoclusters on NaTaO_3_ nanoparticles, producing a library of Cu­(SAC)@NaTaO_3_ or Cu­(SnC)@NaTaO_3_ with controlled amounts of Cu­(SAC) and Cu­(SnC), respectively. *In situ* Electron Paramagnetic Resonance (EPR) spectroscopy was used as a state-of-the-art tool to map the precise configuration of the Cu^2+^ species on NaTaO_3_, as well as the photoinduced electron transfer from NaTaO_3_ to surface-anchored Cu^2+^. Photocatalytic H_2_ production from H_2_O demonstrates that Cu­(SnC)@NaTaO_3_ i.e. decorated with Cu^2+^ subnanoclusters, exhibits significantly superior activity vs their single Cu-atom counterpart, achieving enhanced H_2_ photogeneration of >10,900 μmol/g/h, corresponding to an apparent quantum yield of 1.5%, with no noble metal added as a cocatalyst. EPR data show that Cu^2+^ subnanoclusters in Cu­(SnC)@NaTaO_3_ are ∼300% more efficient electron acceptors compared to Cu^2+^ monomers in Cu­(SAC)@NaTaO_3_. Transmission electron microscopy (TEM), Raman spectroscopy, and X-ray photoelectron spectroscopy (XPS) data reveal that FSP-deposited Cu^2+^ SnC forms a tight interface with the NaTaO_3_ surface, leading to an improved energy-level configuration. Overall, the present data showcase, for this particular photocatalytic system, that Cu subnanoclusters rather than the currently believed single Cu atoms are the preferable cocatalyst in H_2_O photocatalysis by NaTaO_3_. This postulate probably can be verified for other pertinent systems. Moreover, we demonstrate that FSP-made Cu­(SnC)@NaTaO_3_ is a highly promising, noble-metal-free photocatalyst.

## Graphical abstract

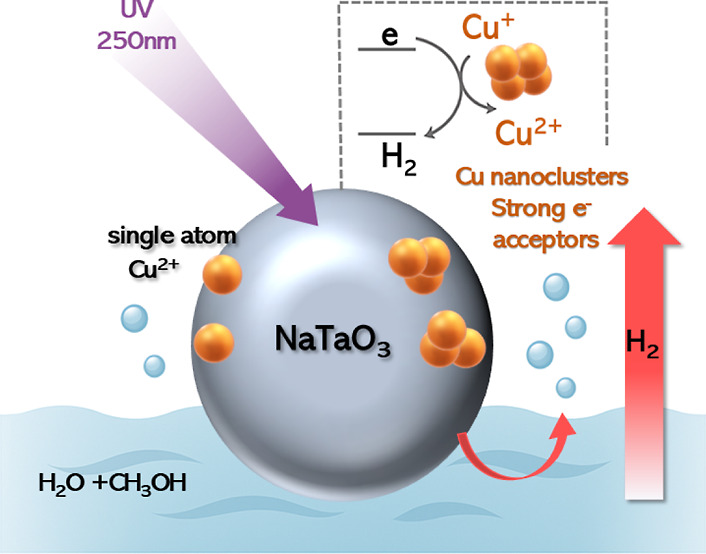

## Introduction

Green hydrogen production can mitigate key environmental and energy challenges that society currently encounters.^1,2^ Currently, among various approaches toward green-H_2_ production, photocatalytic H_2_ production from H_2_O is highly eligible, leveraging sunlight to produce hydrogen from water. A crucial aspect of this technology is the development of efficient photocatalysts with high efficiency and stability.^3,4^ To this end, NaTaO_3_ is a photocatalytic perovskite with promising characteristics for photocatalytic H_2_ production, mainly due to its highly reducing conduction-band edge (*E*_CB_ = −1.06 vs NHE).^5–7^ However, so far, full exploitation of NaTaO_3_ as an H_2_ photocatalyst requires diligent control of fundamental issues typically encountered in photocatalysis,^8,9^ e.g., particle size, bandgap engineering, and selection of optimal cocatalysts. Recently,^10^ we have shown that the challenge of decreasing the particle size of NaTaO_3_ to critical limits can be achieved by flame spray pyrolysis (FSP) technology. This allowed us to produce the smallest so far, NaTaO_3_ of the order of 10 nm, which demonstrated the best so far NaTaO_3_-photocatalytic H_2_ production from H_2_O of 6000 μmol/g/h.^10^ In addition, using Electron Paramagnetic Resonance (EPR) spectroscopy,^11^ we monitored *in situ* the formation and dynamics of photoinduced {holes/electrons}, demonstrating the key role of nanosize, well beyond the known, beneficial effect^10,11^ of enhanced specific surface area.

Apart from the nanosize, modifications of the photocatalyst surface or lattice also have to be tuned in order to enhance photoactivity. In this direction, eligible strategies can include elemental doping,^12–16^ noble metal deposition on the catalyst’s surface,^9,17^ and the introduction of defects or strains.^18,19^ Formation of heterojunction structures^20–22^ can be another strategy to boost photocatalytic performance.

In recent years, within the mandate of greener technologies, replacing noble metals as cocatalysts, earth-abundant non-noble elements, e.g., Cu, Ni, Co, or Sn, have gained ground.^23–29^ Of course, the key challenge is to retain or enhance the performance when the non-noble catalyst is used *in tandem* with the use of the minimal amount of cocatalyst. In this context, Cu is anticipated as a highly promising non-noble (photo)­cocatalyst due to its ability to participate in electron transfer processes via its reversible {Cu^2+^–Cu^1+^}/{Cu^1+^–Cu^0^} redox ability, effectively acting as a trapping site for charge carriers, thus preventing recombination of photogenerated electron–hole pairs.^30,31^

Single atoms dispersed on a semiconductor surface can enhance catalytic activity and selectivity, functioning as single-atom (co)­catalysts (SAC).^32–35^ The benefits of such SACs can be due to their low-coordination environment and, more intriguingly, their ability to modulate electronic states at their interface with the semiconductor.^32–35^ If properly tuned, SACs can facilitate efficient charge transfer between the catalyst and cocatalyst,^36^ which can be further enhanced via strong metal–support interaction (SMSI) phenomena^37–39^ that promote optimal tuning of the electronic properties between the cocatalyst and the photocatalyst.

Nevertheless, challenges remain regarding the optimal amount, aggregation state (monomers or clusters), and redox state of the cocatalyst. As discussed recently by us^40^ and others,^41–44^ control of the aggregation state of the cocatalyst is of key importance to fine-tune the electronic properties, i.e., small nanoclusters form multiple energy levels, while single atoms form single atomic states.^40^ To this end, so far, current literature seems to entail that single Cu atoms can be preferable over Cu clusters, i.e., it is considered that single-atom (co)­catalysts increase the number of active sites due to the dispersion of individual atoms; plus, they can enhance light absorption, and they can improve charge separation and migration kinetics by altering the electronic structure.^45,46^ For example, a {1% Cu–ΤiO_2_} photocatalyst was reported to exhibit a high H_2_ production rate of 101.7 mmol/g/h and a quantum efficiency of 56% at 365 nm.^47^ The authors claimed that single Cu atoms were the origin of this high performance;^47^ however, their EPR spectra in their Figure 4 indicates Cu aggregates, *not* Cu atoms. This issue will be discussed by numerical simulation of the EPR data herein. In another pertinent work, the authors state that they had developed a subnanocluster Cu cocatalyst on a TiO_2_ photocatalyst that achieved H_2_ production of 6832 μmol/g/h, comparable to that of Pt–TiO_2_ (7754 μmol/g/h).^48^ Thus, despite converging evidence that groups of Cu^2+^ atoms can be good cocatalysts for H_2_ photocatalysis, there is currently a lack of convincing understanding of what the preferable Cu configuration is: true single Cu atoms or small Cu clusters or small Cu particles, i.e., Cu formations with elementary lattice periodicity.^40^

Herein, our aim was to clarify this issue by studying the role of {single Cu^2+^ atoms} vs {Cu^2+^ subnanoclusters}, deposited on the NaTaO_3_ particle surface, as cocatalysts for optimal photocatalytic H_2_ production from H_2_O. Therefore, we developed a library of NaTaO_3_-perovskite nanoparticles decorated with various Cu^2+^ configurations. For the synthesis, we used a novel Flame Spray Pyrolysis process, described herein, see scheme in Figure 1, which allows engineering of NaTaO_3_ nanocatalysts decorated with the desired configuration of cocatalysts, in a single step. We underline that the synthesis of small NaTaO_3_, and more generally ABO_3_ perovskite nanoparticles, is challenging since ABO_3_ particles require the simultaneous insertion of A and B cations to form an ABO_3_ lattice at the correct stoichiometry of 1:1:3.^40–49,49–51^ To do this, usually, a high-*T* calcination is typically used in synthesis protocols.^52–54^ Recently, we demonstrated an FSP process that allows the production of highly crystalline small NaTaO_3_ nanoparticles (<15 nm) by adjusting the combustion enthalpy and the high-temperature residence time.^10^ Herein, we used this protocol for FSP production of NaTaO_3_, and then—in the same process—we deposited *in situ* Cu^2+^ atoms via spraying in a low-*T* chamber, see Figure 1. In this way, we obtained {single Cu atoms/NaTaO_3_} or {Cu subnanoclusters/NaTaO_3_} or {CuO particles/NaTaO_3_}. The exact Cu configuration in each material was identified and quantified using electron paramagnetic spectroscopy (EPR).^55–58^ In brief, the EPR spectra of Cu^2+^ ions allow a detailed mapping of the Cu–Cu distance via the dipolar and/or exchange interactions of the spins of Cu^2+^ atoms;^59^ as we showed,^60^ proximal/ but-discrete Cu atoms can be precisely distinguished from Cu clusters based on the experimental EPR spectra and their numerical simulations. Furthermore, analysis of the EPR **g** and **A** tensors of Cu^2+^ ^61^ allows a detailed mapping of the coordination environment of Cu^2+^ atoms on particle surfaces. In this context, herein, we exemplify the use of EPR spectroscopy as a state-of-the-art tool to address the details of the {single Cu atoms} vs {Cu clusters} vs {small CuO particles} in connection to their cocatalysis role in H_2_O/H_2_ photocatalysis on the NaTaO_3_. We underline that, herein, the notion of {small CuO particles} refers to cases of small CuO <2 nm where X-ray diffraction (XRD) cannot easily detect them, while EPR can. Furthermore, with our focus being on photocatalytic H_2_ production, experiments were conducted under light irradiation, where *in situ* EPR was used to monitor the electron transfer from NaTaO_3_ to the surface-anchored Cu^2+^ species.

**Figure 1:**
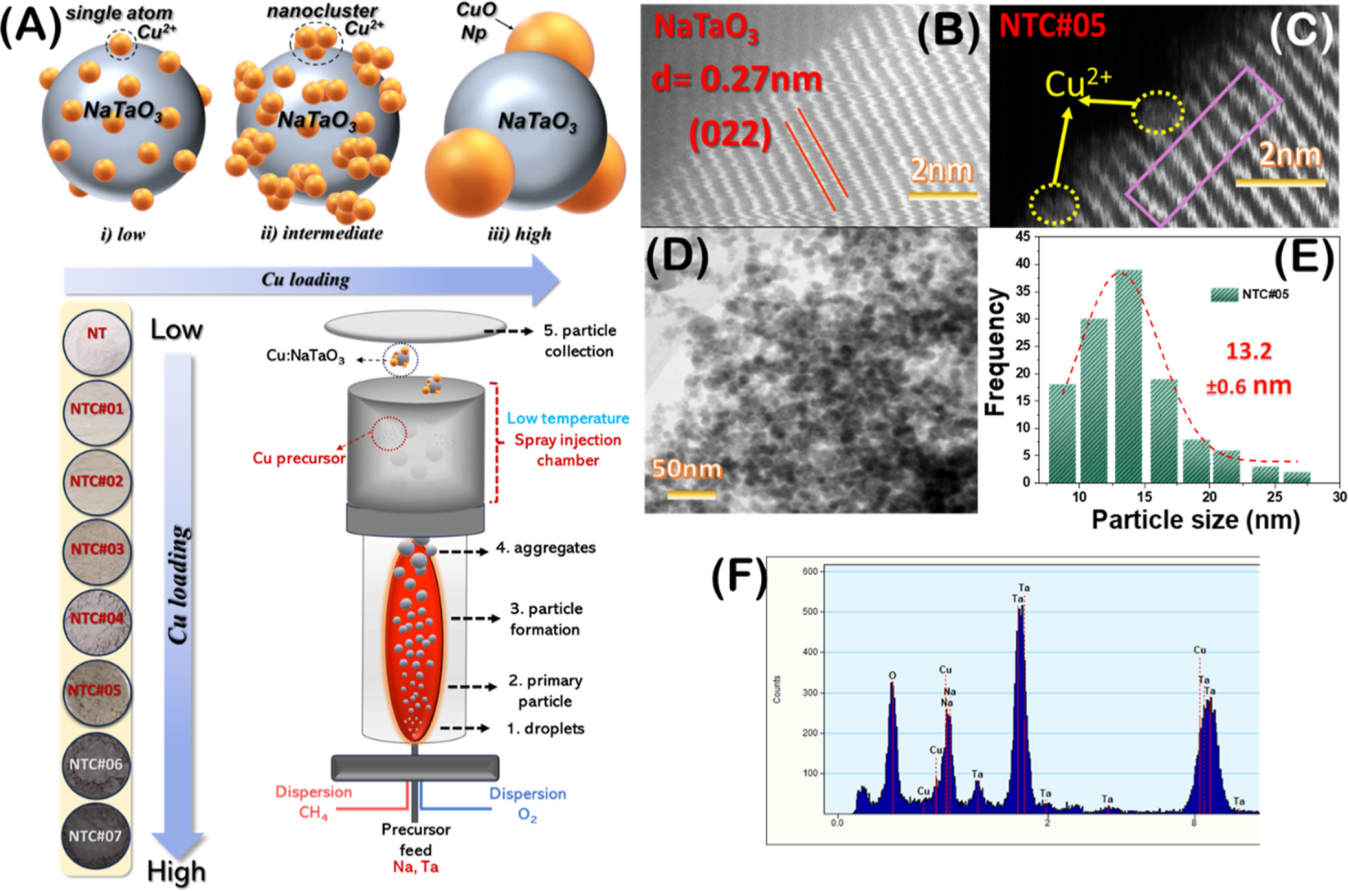
(A) Schematic illustration of the synthesis of NTC nanoparticles, (B) TEM image of pristine NaTaO_3_, (C,D) TEM images of NTC#05 in different magnifications, (E) statistic size distribution of NTC#05, and (F) EDS spectrum of NTC#05.

Overall, specific aims of the present work are as follows: (i) to use FSP to produce small NaTaO_3_ nanoparticles and decorate them with either {single Cu^2+^ atoms} or {Cu^2+^ subnanoclusters} or {small CuO nanoparticles} in one step, (ii) to use *in situ* EPR spectroscopy to determine quantitatively the Cu formations, and (iii) to study the role of the Cu configurations as cocatalysts in photocatalytic H_2_ production without any noble metal.

## Methods

### Synthesis of NaTaO_3_ by Flame Spray Pyrolysis

The precursor solutions containing anhydrous tantalum­(V) chloride (99.9%-Ta, STREM) and sodium 2-ethylhexanoate (98%, TCI) were both dissolved in ethanol (99%, Emsure). NaTaO_3_ particles were produced according to the FSP protocol we reported recently.^10^ In brief, 0.05 M TaCl_5_ and 0.3 M C_8_H_15_NaO_2_ were dissolved in ethanol, and the excess Na salt was removed from the precursor solution by centrifugation. The flame spray pyrolysis (FSP) process for producing the final particles included a dispersion oxygen flow rate of 5 L/min (Linde 99.999%) and a precursor flow rate of 5 mL/min. The initial flame was ignited using premixed O_2_ and CH_4_ at flow rates of 2 and 1 L/min, respectively, contained within a 20 cm metal tube positioned at 30 mm over the nozzle. The generated particles were deposited on a glass microfiber filter with a binder (Albet Labscience GF_6_257) using a vacuum pump (BUSCH), and the powder was collected by scrubbing it off the filter. The nanomaterials were then stored in glass vials under an inert argon atmosphere until use.

### In Situ Cu Deposition on NaTaO_3_ (NTC Materials)

The characterisitcs of the synthesized Cu-decorated NaTaO_3_ nanomaterials, herein codenamed as NTC, are listed in Table 1. The FSP process, depicted in Figure 1, was based on two subsequent steps: first, the formation of the NaTaO_3_ nanoparticles by FSP as detailed hereabove. The formed NaTaO_3_ nanoparticles were vertically directed through a spraying chamber, where a solution of Cu­(NO_3_)_2_ in ethanol (99%, Emsure) was sprayed by three sprayers located 35 cm above the burner nozzle. The spraying chamber was cooled by a cold N_2_ stream injected radially inside the chamber. This setup exposes the stream of preformed NaTaO_3_ particles to a low-temperature stream <300 °C, monitored by a thermocouple, to the {Cu­(NO_3_)_2_/ethanol} cloud. By adjusting the NaTaO_3_ flow, the {Cu­(NO_3_)_2_/ethanol} amount, and the cooling N_2_, we could deposit controlled amounts of Cu on the NaTaO_3_. Based on screening experiments, we have produced {Cu/NaTaO_3_} with nominal Cu-loading of 0.05, 0.1, 0.5, 1, 2.5, 5, and 10% w:w, Cu:Ta. The FSP parameters are listed in Table 1. The produced materials, also listed in Table 1, are condemned as NTC#01 for 0.05% Cu:Ta, NTC#02 for 0.1% Cu:Ta, and so on. The control NaTaO_3_ is codenamed as NT.

**Table 1:** Structural Characteristics of Cu@NaTaO_3_ Nanoparticles, *d*_BET_, *d*_XRD_, SSA, and *E*_g_

material	characteristics	Cu^2+^ loading (% w/w)	*d*_XRD_ (nm) (±0.5)	Specific Surface Area (m^2^/g) (±0.5)	*d*_BET_ (nm) (±0.5)	band gap *E*_g_ (eV) (±0.1)
NT	NaTaO_3_		21	44	19	4.0
NTC#01	Cu^2+^ deposition on NaTaO_3_	0.05	14	57	14.7	3.9
NTC#02	//	0.1	14	58	14.5	4.0
NTC#03	//	0.5	14	55	15.3	4.0
NTC#04	//	1	13	62	13.5	4.0
NTC#05	//	2.5	14	56	15	4.0
NTC#06	//	5	15	37	22	4.2/1.5
NTC#07	//	10	16	33	25	4.2/1.5

#### Characterization Techniques

##### Powder X-ray Diffraction

Powder X-ray diffraction (XRD) measurements were taken at room temperature using a Bruker D8 ADVANCE 2theta diffractometer with copper radiation (Cu Kα, λ = 1.5406 Å) and a secondary monochromator operating at 36 kV and 36 mA. Crystal size was determined using the Scherrer formula. The particle crystal size *d*_XRD_ was calculated with the Scherrer equation (eq 1).

1 $$d_{XRD}=\frac{K\lambda }{\left(FWHM\right)\times \cos\theta }$$

where *K* = 0.9, λ = 1.5418 Å, and FWHM is the full-width at half-maximum of the XRD peak.

##### Transmission Electron Microscopy

Transmission electron microscopy (TEM) was used to investigate the morphology, nanostructure, and chemical composition of nanoparticles with an FEI Titan 80–300 S/TEM microscope operating at an accelerating voltage of 300 kV. Scanning TEM (STEM) images were captured with a beam convergence semiangle of 21.5 mrad. Sample preparation involved sonicating powdered samples in ethanol and depositing a single droplet of the homogeneous suspension onto a TEM copper grid covered with a lacey carbon film. Before observation, the samples were treated in argon plasma for 3 s using a Fischione Instruments 1020 Plasma Cleaner to eliminate any organic contamination.

##### Diffuse Reflectance Spectroscopy

Diffuse reflectance spectroscopy (DRS) experiments were performed using a PerkinElmer Lambda 35 spectrophotometer. This instrument employs a halogen lamp and a deuterium lamp to span the wavelength range of 190 to 1100 nm, with the transition from the halogen lamp to the deuterium lamp occurring at approximately 380 nm.

##### Raman Spectroscopy

Raman spectroscopy was conducted using a HORIBA-Xplora Plus spectrometer equipped with an Olympus BX41 microscope. A 785 nm diode laser served as the excitation source with the laser beam focused on the sample via the microscope. Each powder material was gently pressed between two glass plates to form a pellet-like structure before measurement.

##### X-ray Photoelectron Spectroscopy

X-ray photoelectron spectroscopy (XPS) data were obtained with a surface analysis ultrahigh vacuum system (SPECS GmbH) featuring a twin Al–Mg anode X-ray source and a multichannel hemispherical sector electron analyzer (HSA Phoibos 100). The base pressure was between 2 and 5 × 10^–9^ mbar. All XPS measurements used a monochromatized Mg Kα line at 1253.6 eV and an analyzer pass energy of 20 eV. Binding energies were referenced to the C 1s peak of contaminant carbon at 284.5 eV, and peak deconvolution employed a Shirley background.

##### Brunauer–Emmett–Teller Analysis

Brunauer–Emmett–Teller (BET) adsorption–desorption isotherms were recorded at 77 K with a Quantachrome NOVA touch LX2, following outgassing at 80 °C for 5 h under vacuum. The specific surface area (SSA) was calculated using adsorption data points in the relative pressure *P*/*P*_0_ range of 0.1–0.3. The SSA-equivalent diameter *d*_BET_ of the particles was determined using the following equation:

2 $$d_{BET}=\frac{6000}{SSA\times \rho_{NaTaO_{3}/Ta_{2}O_{5}}}$$

where the density of the particles was ρ_NaTaO_3__ = 7.129 g/cm^3^ and ρ_Ta_2_O_5__ = 8.2 g/cm^3^, according to the corresponding crystal structure.

##### Electron Paramagnetic Resonance Spectroscopy

EPR spectra were recorded using a Bruker ER200D spectrometer at liquid-nitrogen temperatures (77 K) equipped with an Agilent 5310 A frequency counter operating at the X-band (∼9.6 GHz) with a modulation amplitude of 10 G peak to peak. The spectrometer is controlled with custom-made software based on LabView. To obtain an adequate signal-to-noise ratio, each spectrum has an average of 10–20 scans.

### Theoretical Analysis of the Cu^2+^ EPR Signals

The simulation of monomeric Cu^2+^ (*S* = 1/2, *I* = 3/2) EPR spectra was performed using a spin Hamiltonian ([Disp-formula eq3]):

3 $$H=\beta_{e}B\mathrm{g̃}S-\beta_{n}g_{n}BI+SAI$$

where *g*_n_ represents the nuclear *g*-factor, while β_e_ and β_n_ denote the Bohr and nuclear magnetons, respectively. The first and second terms correspond to the electronic and nuclear Zeeman interactions, respectively, where **B** is the external magnetic field expressed in the principal axes system of the *g*-matrix. The final term arises from the copper hyperfine interaction, with the nuclear spin of copper *I* = 3/2 for both naturally occurring isotopes ^63^Cu (69.09% abundance) and ^65^Cu (30.91% abundance). The hyperfine coupling tensors (*A*-tensors) for Cu^2+^ were modeled as axially symmetric (*A*_*xx*_ = *A*_*yy*_ ≠ *A*_*zz*_), consistent with the typical electronic structure of d^9^ systems in approximately tetragonal ligand fields. In such environments, the unpaired electron resides predominantly in the d_*x*_^^2^^_–*y*_^^2^^ orbital, giving rise to axial symmetry in both the *g*- and *A*-tensors. While slight deviations from axiality may exist due to structural distortions or strain, particularly in surface-bound species, these are expected to be minor and are not resolvable. Numerical simulations were performed using EasySpin, a comprehensive, open-source MATLAB toolbox^62^ for EPR spectroscopy simulations. The simulations employed a Gaussian line shape with a typical line width parameter of 5 G. For the simulation of the Cu clusters, we have used a multispin Hamiltonian assuming *N* = 2 up to 10 Cu (*S* = 1/2, *I* = 3/2) spins, taking into account the information from the HR-TEM data also.

In each case of 2 Cu^2+^ atoms, the spin Hamiltonian was of the form

4 $$H^{ab}=\beta_{e}Bg^{a}S^{a}+\beta_{e}Bg^{b}S^{b}+S^{a}A^{a}I+S^{b}A^{b}I+S^{a}D^{ab}S^{b}$$

where the index “*a*” signifies the first Cu^2+^ atom and “*b*” a second Cu^2+^ atom. The strength of interaction between Cu^*a*^ and Cu^*b*^ is described by tensor-**D** ^**ab**^ via the term **S** ^a^ **D** ^**ab**^ **S** ^**b**^ in eq 4. In the case of 3 Cu^2+^ atoms, eq 4 can be rewritten as

5 $$H^{abc}=H^{ab}+\beta_{e}Bg^{c}S^{c}+S^{c}A^{c}I+S^{a}D^{ac}S^{c}+S^{b}D^{bc}S^{c}$$

where *H* ^ab^ is the two-Cu spin Hamiltonian of eq 4. For *N* > 3 Cu atoms, a similar algorithm was used. The key concept is that we allow pairwise Cu–Cu interaction through the **D** ^**ij**^ spin–spin tensor, whose diagonal elements reflect the strength of the Cu–Cu coupling. For simplicity, we assumed that all **D** ^**ij**^ tensors are diagonal in the same frame of reference, i.e., the *Z-*axis was assumed to be determined by the external Zeeman *B*-field. In this approximation, we ignore off-diagonal *D* ^*ij*^ elements.^63^ Our numerical calculations (see Figures 4 and S5 in Supporting Information) show that the *D* ^*ij*^ values listed in Table 2 can reproduce the observed EPR-line shape trends. Specifically for two Cu ions, principal values of *D* ^*ab*^ in the order of <20 G (see Figure S5 in Supporting Information) reproduce the line-broadening of the EPR signals with no fundamental shifts of spectra reshaping. Then, for *D* ^*ab*^ > 50 G (Figure S5 in Supporting Information), we observe a progressive broadening and loss of the hyperfine resolution, in accordance with EPR theory,^63^ where the spin–spin coupling *D* ^*ab*^ becomes ≥*A*-tensors principal value. Then, when more Cu spins are coupled, there is a smear out of all hyperfine features, plus a characteristic narrowing of the overall EPR spectrum. These results are in agreement with the original EPR spectra of Fujiwara et al.^64^ for *n*-Cu atoms, where the loss of hyperfine features and appearance of a doublet-like EPR spectrum indicate that more than *N* > 10 Cu atoms are closely interacting. In this way, in the present case, our library of theoretical EPR spectra, Figure S5 in the Supporting Information, allowed us to clearly distinguish Cu monomers from Cu subnanoclusters.

**Table 2:** Spin-Hamiltonian Parameters and Cu-Population Analysis Derived by Numerical Simulations of the EPR Spectra for the Cu/NaTaO_3_ Materials

material	monomeric Cu^2+^		Cu^2+^-clusters		
	[*g*_*x*_, *g*_*y*_, *g*_*z*_] ± 0.002 [*A*_*z*_] (Gauss ± 5)	% of total Cu-content (±5%)	[*g*_*x*_, *g*_*y*_, *g*_*z*_] ± 0.002 [*A*_*z*_] (Gauss ± 5)	*N* (number of atoms) per cluster [*D*] (Gauss ± 1)	% of total Cu-content (±5%)
NTC#01	[2.068 2.085 2.364] [177]	77	[2.041 2.098 2.382] [142]	*Ν* = 4 [16]	23
NTC#02	[2.055 2.086 2.365] [171]	36	[2.059 2.09 2.373] [2, 2, 161]	*Ν* = 4 [22]	64
NTC#03	[2.07 2.07 2.365] [168]	13	[2.08 2.08 2.365] [157]	*Ν* = 8 [58]	87
NTC#04	[2.074 2.069 2.359] [151]	9	[2.081 2.082 2.368] [140]	*Ν* = 8 [60]	91
NTC#05	[2.065 2.073 2.363] [161]	6	[2.076 2.092 2.390] [140]	*Ν* = 8 [53]	94
NTC#06	[2.075 2.073 2.364] [163]	3	[2.09 2.09 2.378] [153]	*Ν* = 10 [54]	97
NTC#07	[2.075 2.085 2.364] [163]	0	[2.010 2.011 2.378] [152]	*Ν* = 10 [63]	100

For completeness, we notice that in our experimental EPR spectra, we did not resolve evidence for *g* ∼ 4 Δ*ms* = 2 EPR transitions.^63^ In principle, these “semiforbidden” EPR transitions could be resolved in small Cu dimers or trimers^60–63^ with well-defined geometry and low line broadening. In the present case, the inherent heterogeneity of the electronic microenvironment and the strong line-broadening effects prohibit reliable detection of these *g* ∼ 4 EPR signals.

#### Photocatalytic H_2_ Evolution from H_2_O Splitting

Photocatalytic experiments were conducted in a double-walled photochemical reactor with a total volume of 340 mL at room temperature (25 °C) regulated by a circulation chiller cooling system. A 250 W mercury lamp (UV irradiation) was used as the light source, positioned at the geometric center of the photoreactor within a quartz immersion well. The irradiation power, measured in situ with a power meter (Figure S7) at an average experimental distance of 3 cm, was 0.14 W/cm^2^. Following the catalyst mass optimization shown in Figure S8 in the Supporting Information, 69 mg of catalyst was suspended in 220 mL of triple-distilled H_2_O and 55 mL of methanol (20% v/v) for each experiment. A gas chromatography system equipped with a thermal conductivity detector (TCD) (Shimadzu GC-2014, Carboxen 1000 column, Ar carrier gas) was used to identify and quantify the produced gases.

## Results

### Flame Spray Pyrolysis (FSP) Synthesis of Cu@NaTaO_3_ Nanoparticles: Interfacial Coupling of Cu on NaTaO_3_

The FSP-reactor setup, described in Figure 1A, allows a controlled amount of Cu atoms to be deposited on the NaTaO_3_ in the low-*T* injection chamber via increasing the concentration of the Cu atoms in the low-*T* spray precursor. Decoration with Cu atoms can be visually observed; i.e., the white, pristine NaTaO_3_ (NT) progressively acquires a gray tint, see photos on the side of Figure 1A. HRTEM images of Figure 1B show high-quality (002) Miller planes with a lattice spacing of 0.27 nm. We see that high-crystallinity NaTaO_3_ has been achieved with no evidence of lattice distortion or amorphous regions, not even at the edges of the particles.

In comparison, the HRTEM image for the Cu-loaded NTC#05 sample in Figure 1C reveals regions with resolvable distortion of crystallographic planes, see marks in Figure 1C. Analysis of HRTEM images reveals that the Cu-loaded NTC#05 had a NaTaO_3_ particle size of 13.2 ± 0.6 nm, see Figure 1E, in accordance with XRD (Figure 3A).

Atomic mapping HR-STEM, Figure 2, confirms that our FSP technology allows fine control of the surface-deposited Cu species. Specifically in Figure 2A,B, we observe that [i] at both low Cu loading (NTC#02) and higher loading (NTC#05), the Cu species are homogeneously distributed over that NaTaO_3_ surface, and [ii] in the low-Cu NTC#02 material, the Cu atoms are dispersed with no evidence for cluster formation, while in the NTC#05 material, Cu clusters are formed. These Cu clusters have sizes below 1 nm and are quasi-homogeneously distributed over the NaTaO_3_ surface at cluster–cluster distances of the order of a few Angstroms.

**Figure 2:**
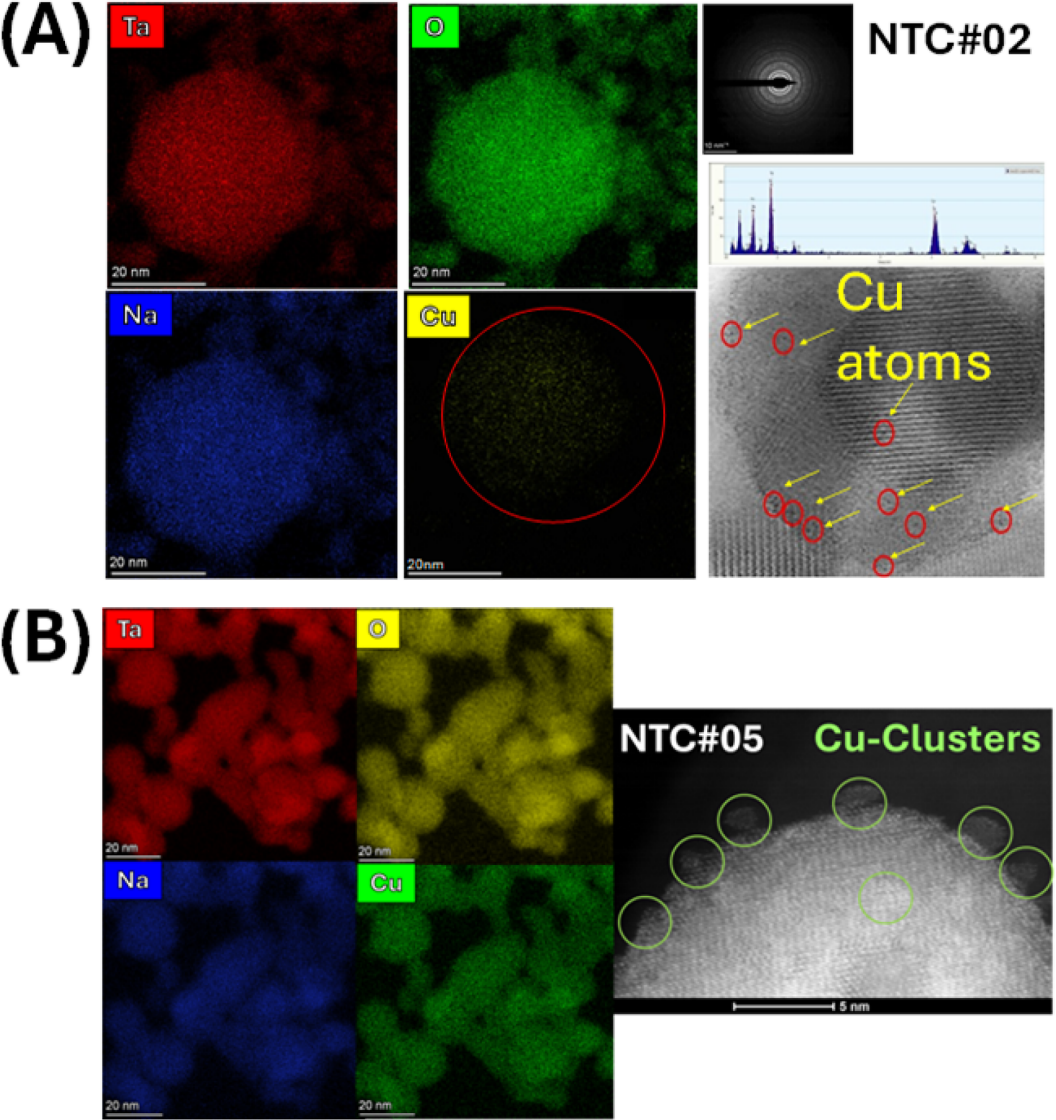
Atomic mapping HR-EDX/STEM images for (A) low-Cu-loading NTC#02 and (B) high-Cu-loading NTC#05 materials. In each panel, the color codes mark the Na, Ta, O, and Cu, respectively. High-resolution images at the right side of each allow visualization of the mode of dispersion of Cu moieties, i.e., monomeric Cu atoms in (A) and Cu clusters in (B).

Raman data that we analyze hereafter confirm these distortions caused by the deposition of the Cu entities on the NaTaO_3_. In the same context, surface and lattice planes of NaTaO_3_ particles in NTC#05 display clear signs of structural distortion, accompanied by small, cluster-like features, marked by the purple frame and yellow circles in Figure 1C, unlike the pristine NaTaO_3_, which remains structurally intact. To further peer into the electronic/redox configuration of the Cu-moieties, hereafter, electron paramagnetic resonance (EPR) spectroscopy was utilized as a state-of-the-art spectroscopy for detecting Cu^2+^ monomers and subnanoclusters, given its exceptional sensitivity to local magnetic environments.

Overall, the significance of these data is that they show that the FSP-deposited Cu entities on NaTaO_3_ are strongly anchored on the surface, i.e., a strong metal–support interaction (SMSI) occurs. This is due to the FSP process, where the Cu deposition occurs inside the hot stream of the NaTaO_3_ particles that are formed right prior to the Cu deposition chamber, see Figure 1A. Controlling the amount of sprayed Cu and the deposition temperature *T* ^deposition^ allows control of the Cu configuration, i.e., low Cu amount/low *T* ^deposition^ allows monomeric Cu atoms, while progressively increased Cu amount/higher *T* ^deposition^ allows Cu clusters and eventually small Cu particles to be deposited on NaTaO_3_.

The XRD data (Figure 3Α) confirm the formation of highly crystalline orthorhombic (*Cmcm*) NaTaO_3_ (PDF 01-085-5477). Samples with low or intermediate Cu loadings, i.e., NTC#01 to NTC#03, do not display any resolvable XRD peaks associated with Cu or CuO, whereas high copper loading in NTC#07 leads to the formation of small CuO particles with *d*_XRD_ = 2–3 nm, identified by the PDF 01-084-4316.

**Figure 3:**
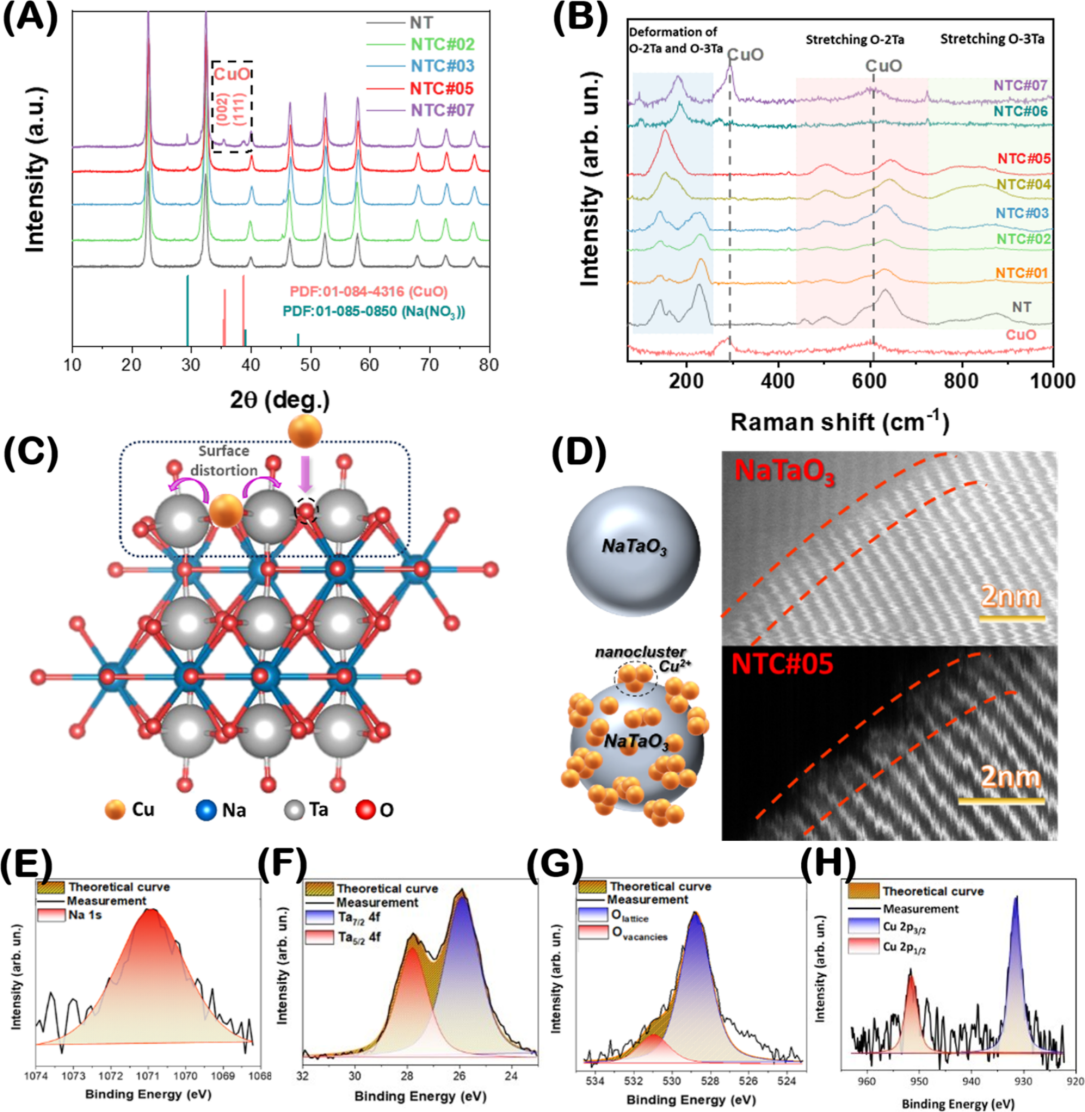
(A) XRD patterns of NT and NTC nanomaterials, (B) Raman spectra of NT and NTC nanomaterials, (C) schematic illustration of the nanoparticle’s surface distortion caused by Cu^2+^ loadings, (D) HRTEM images of NT and NTC samples showing the surface distortion in the latter, and (E–H) XPS spectra of NTC#05.

The XRD crystal sizes of the nanoparticles (*d*_XRD_) calculated with Scherrer eq 1 are presented in Table 1. The *d*_XRD_ of pristine NT was 21 nm, and after Cu loadings, it showed a remarkable decrease to 14 nm. This result is in agreement with the particle size distribution (*d*_TEM_) calculated at 13.2 ± 0.6 nm by TEM images.

Simultaneously, the specific surface area (SSA) was increased from 44 to approximately 60 m^2^/g, see Table 1, which further confirms the decrease of the nanoparticle’s size when Cu was sprayed. As a control, when we sprayed ethanol solvent only with no Cu content, the NaTaO_3_ *d*_XRD_ was decreased to 14 nm (not shown). This demonstrates that the sprayed Cu solution inhibits the NaTaO_3_ growth, as expected in FSP when a cooling agent is cooling the flame. In the case of the high Cu loadings (NTC#06, NTC#07), SSA was 37 and 33 m^2^/g, respectively (see Table 1), without a significant change in the NaTaO_3_ size. For completeness, comparison of the *d*_BET_, calculated via eq 2 and listed in Table 1, shows that in low-Cu-loaded materials, NTC#01–05 *d*_XRD_ ∼ *d*_BET_, which indicates that there are no strong-sintering effects of pore-filling events. In contrast, for NTC#06 and NTC#07, *d*_XRD_ ≫ *d*_BET_, which evidence that deposition of small CuO particles results in pore-filling in Cu@NaTaO_3_.

Raman spectra of pristine NaTaO_3_ and Cu-decorated nanoparticles are shown in Figure 3B. According to literature, the Raman of NaTaO_3_ in the range of 150–1000 cm^–1^ can be conceptually divided into three types of modes:^10^ the modes 150–260 cm^–1^ are attributed to the deformation of O–2Ta or O–3Ta,^10^ while the bands at ∼410–720 cm^–1^ correspond to stretching modes of O–3Ta.^10^ Furthermore, the bands >720 cm^–1^ are ascribed to the stretching vibration modes of O–2Ta.^65,66^ Herein, the detection of the bands below <250 cm^–1^, Figure 3B, in the spectra of all samples validates that the nanomaterials are free of amorphous regions.^10^ This confirms that our advanced FSP setup produces high-crystallinity NaTaO_3_ nanoparticles, as we reported originally for a simpler FSP setup.^10^ Comparing the Raman for pristine NaTaO_3_ in Figure 3B with the spectra of the Cu-decorated nanomaterials, we see that the low-copper content (<1% Cu) NTC#01,2,3 do not exhibit apparent differences. However, as the Cu content increases >1%, drastic changes occur primarily at 150 and 260 cm^–1^, where a gradual broadening of these two characteristic peaks is observed. Upon further increase in Cu content, the two peaks tend to broaden/merge toward 154 cm^–1^, while, ultimately, in NTC#06 and NTC#07 (with high Cu content of 5 and 10%), the merged band of 154 cm^–1^ is replaced by characteristic bands of CuO at ∼210 and 610 cm^–1^, indicating the formation of CuO nanoparticles.^67,68^ These results are in agreement with XRD, which, however, resolved only the CuO for NTC#07. The broadening and redshifting of the O–Ta vibrational modes (150–260 cm^–1^ range) with increasing Cu loading directly correlate with Cu aggregation from monomers to clusters (EPR data, Table 2). This suggests that lattice distortion likely results from strain or bonding variations introduced by the Cu incorporation. Thus, Raman and XRD show that for Cu loadings above 5%, we have the formation of small CuO particles of sizes 2 to 3 nm.

Overall, the present HRTEM, XRD, and Raman show clearly that deposition of Cu on the NaTaO_3_ surface by our low-*T* FSP setup allows controlled growth of the Cu entities, causing significant disorder of the NaTaO_3_ bonds, as illustrated in Figure 3C. That conclusion suggests that during the present NaTaO_3_ FSP process, the NaTaO_3_ particles are not yet completely formed when they encounter the droplets of the Cu precursor. This interaction leads to a semidistorted surface, as evidenced by HRTEM and Raman data, e.g., more clearly for NTC#05 vs NaTaO_3_. Consequently, this structural distortion leads to strong metal–support interactions (SMSIs) between the catalyst and cocatalyst, which, as we show in the following, is a key benefit improving their photocatalytic H_2_-production performance.

Τo peer further into the surface properties of the NTC#05 material, XPS measurements were carried out. The XPS spectra in Figure 3Ε–Η verify the presence of Cu, as well as Na, Ta, and O elements without other impurities, excluding adventitious carbon-based contaminants. Specifically, the characteristic Na 1s binding energy of 1071 eV, shown in Figure 3Ε, confirms the existence of Na atoms and their incorporation into the Ta_2_O_5_ lattice, forming the NaTaO_3_ structure.^21^ According to the Ta 4f spectrum in Figure 3F, two prominent bands at 25.9 and 27.8 eV are observed, characteristics of Ta^5+^ in NaTaO_3_.^69^ Additionally, the Ta-XPS spectrum shows the distinct Ta_5/2_^5+^ and Ta_7/2_^5+^ peaks, while reduced Ta states, e.g., Ta^4+^, Ta^3+^, and/or Ta^0^, were absent in the XPS spectra.^21^ The O-XPS spectrum (Figure 3G) presents two characteristic peaks at 531 and 529 eV assigned to surface hydroxyl groups and the O–Ta bonds of the crystal, respectively. Finally, in Figure 3H, the two main bands at 932 and 952 eV correspond to the distinct 2p_3/2_ and 2p_1/2_ of Cu^2+^, Cu^1+^, and/or Cu^0^, respectively;^70,71^ however, due to the low Cu content, the Cu-XPS signal has low resolution, and further analysis is not possible. For that reason, EPR spectroscopy was employed to study the Cu species more accurately. Overall, the present XPS analysis confirms that there are no oxygen defects or reduced-Ta atoms in the FSP-made NaTaO_3_ lattice and further validates the presence of copper in NTC#05 material.

### Electron Paramagnetic Resonance (EPR) Analysis of the Cu Entities: Analysis of Singe Cu Atoms vs Cu Subnanoclusters

The 77 K X-band EPR spectra for the NTC materials are presented in Figure 4A. The EPR spectra show a progressive broadening on going from the low-Cu-loading NTC#01 toward the high-Cu-loading NTC#07. The EPR spectrum for NTC#01 (spectrum in Figure S5 in Supporting Information) is a typical EPR spectrum for monomeric Cu^2+^ (*S* = 1/2, *I* = 3/2) atoms^60,61^ on oxide surfaces or zeolites.^56^ Typically, the *I* = 3/2 nuclear spin of Cu^2+^ gives rise to the quadruplet/four hyperfine peaks that correspond to the *A*_//_-component of the hyperfine tensor, see eq 3, coupled to the *g*_//_-value of the Cu-*g* tensor, as marked in Figure 4B. As we discussed hereabove, in the section for theoretical simulation of the EPR, see also the library of the theoretical EPR spectra in Figure S5 of the Supporting Information; proximal Cu–Cu pairing induces broadening of the EPR spectrum, which, for an increasing number of proximal Cu atoms and their coupling strength, results in severe spectral broadening and progressive loss of the resolution of the hyperfine quadruplet, i.e., compare the EPR spectra in Figure 4B for NTC#02 vs Figure 4C for NTC#04 and Figure 4D for NTC#07, respectively.

**Figure 4:**
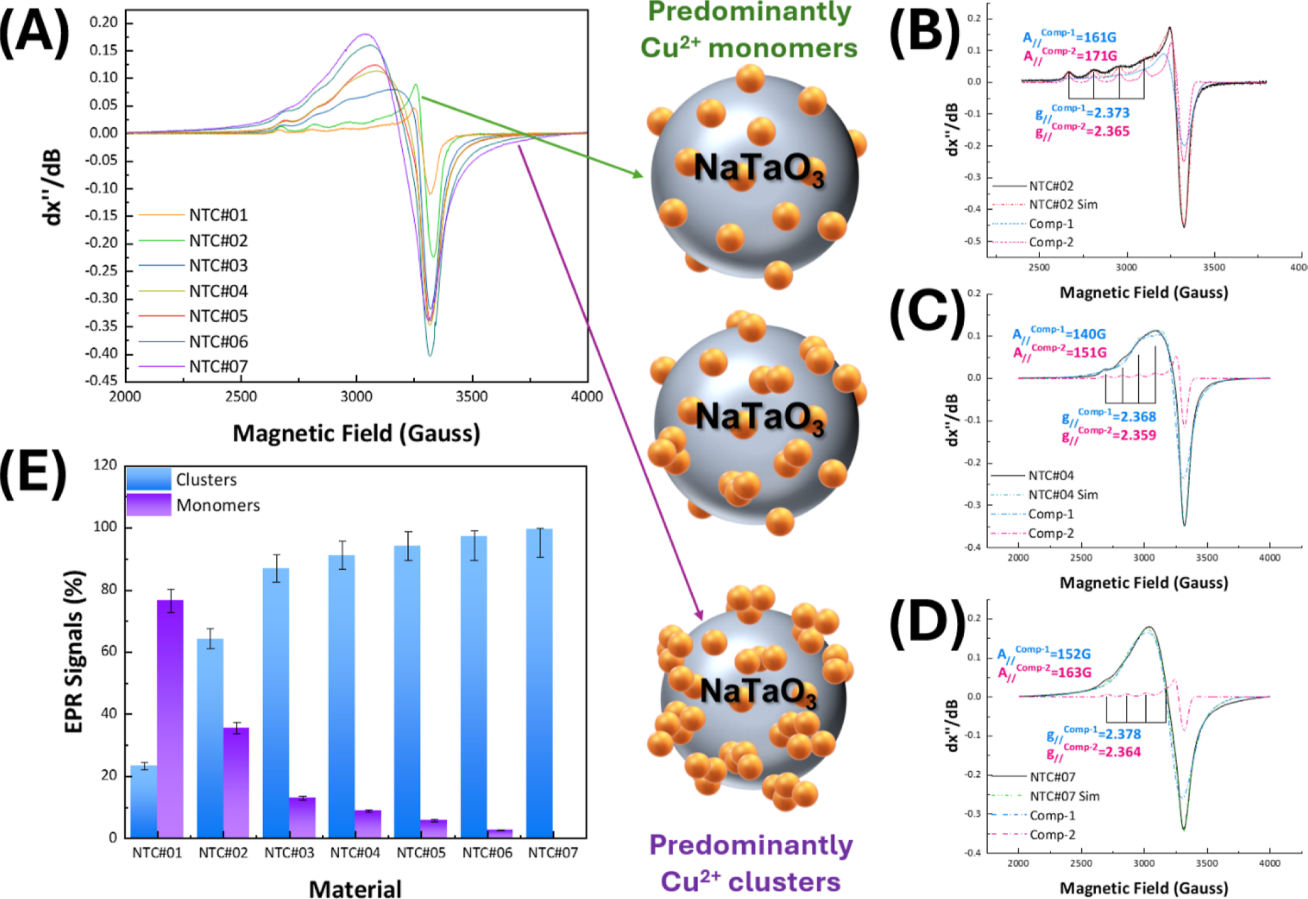
(A) Experimental EPR spectra recorded at 77 K, for the NTC perovskites with the arrows pointing at the extreme cases of Cu^2+^monomers and Cu^2+^ clusters. (B–D) Experimental (solid lines) and simulated EPR spectra (dotted lines) for the materials NTC#02, NTC#04, and NTC#07, respectively. (E) Quantified (%) EPR signals. By increasing the Cu^2+^ surface loading, we observe an increase in the Cu^2+^ cluster component.

Based on our numerical simulations, we have deconvoluted the experimental EPR spectra as shown in Figure 3B–D as follows: each spectrum was simulated in full detail as a superposition of [monomeric Cu^2+^ atom]-EPR plus [Cu clusters]-EPR, using the spin-Hamiltonian parameters listed in Table 2 (see dotted lines in Figure 4B–D). As shown in Figures 4B–D and S5 in the Supporting Information, the simulated spectra mostly replicate the experimental data; i.e., a deviation is observed as shown in Figure 4E, which is within the estimated experimental uncertainty and reflects the inherent complexity of Cu clustering and its influence on spectral features. Despite this, the simulations reliably reproduce the key spectral characteristics, such as *g*-values, line shapes, and hyperfine splitting, demonstrating their overall accuracy and robustness in capturing the underlying electronic structure.

According to the well-established quantitative-EPR methodology,^72^ the double integral of each EPR subspectrum allows calculation of the population of Cu, based on proper calibration of Cu-EPR data, see Figure S5 in Supporting Information. According to this analysis, the percentage of [monomeric Cu^2+^ atom] vs [Cu clusters] presented in Figure 4E shows that the lowest-Cu-loading material NTC#01 contains primarily ∼80% Cu^2+^ monomers, with 20% small Cu clusters (*N* = 2,3). Material NTC#02 shows a rapid increase of the [Cu clusters] vs [monomeric Cu^2+^ atom], i.e., 65/35%. Then, from NTC#03 to NTC#06, there is a dramatic increase of Cu-clusters at the expense of the monomeric Cu atoms. Finally, material NTC#07, which according to the XRD shows the formation of small CuO particles, contains practically zero monomeric Cu atoms. Thus, the EPR spectra and their diligent numerical simulations demonstrate that our low-*T* FSP process allows a controllable deposition of Cu moieties on NaTaO_3_ from mainly monomeric Cu atoms to small Cu clusters and small CuO particles.

Further analysis of the *g*- and *A*-tensors listed in Table 2 for NTC#02 shows the mononuclear Cu^2+^ atoms have two slightly different but distinct coordination environments (I, II), i.e., one type of Cu^2+^ atoms with ^I^Cu^2+^ (*A*_//_ = 171G, *g*_//_ = 2.365) and the second ^II^Cu^2+^ (*A*_//_ = 161G, *g*_//_ = 2.373). According to the Peisach and Blumberg analysis,^60,73^ these *A*_//_ and *g*_//_ show that both types of isolated Cu^2+^ atoms are coordinated by 2-O or 3-O atoms, indicating that they are all located on/or very near the surface of NaTaO_3_. The ^I^Cu^2+^ (*A*_//_ = 171G, *g*_//_ = 2.365) atoms are more strongly anchored on the NaTaO_3_ surface than ^II^Cu^2+^ (*A*_//_ = 161G, *g*_//_ = 2.373). Interestingly, this analysis reveals that at increasing Cu loading, the details of the copper coordination vary dynamically together with the clustering phenomena.

Overall, the preset EPR data and their analysis provide a detailed, quantitative mapping of the evolution of the conformation of the Cu moieties on the NaTaO_3_ particles, see the visualization scheme in Figure 4: NTC#01, with low Cu loading, has predominantly monomeric Cu^2+^ ions anchored on or very near the surface O atoms of NaTaO_3_. NTC#02 has smaller, but non-negligible, populations of monomeric Cu^2+^ atoms, while Cu clusters form at significant populations. Then, NTC#03 and NTC#04 have predominantly Cu clusters, while NTC#05 and #06 are practically decorated exclusively with Cu clusters on their surface. The clusters on these particles account for a limited number of Cu atoms; thus, they are subnanoclusters. Finally, NTC#07 shows the formation of large Cu clusters, some of them forming small-period lattices detectable also by XRD.

At this point, we would like to underline that the quantitative Cu content in such low Cu loadings can be determined by EPR, by the techniques used herein. XPS in principle can give a ratio of Cu/M; however, due to the low Cu content, the Cu-XPS signal has low resolution, and further analysis is not possible. Raman does not provide quantitative information, while XRD does not detect the low-content/noncrystalline Cu. EPR detects quantitatively all Cu that is in the 2+ oxidation state. Since in our FSP process we use oxygen-rich production conditions, 100% of the copper is 2+, i.e., 100% detectable by EPR. As we show in the light-dependent EPR analysis, Figure 7, hereafter, these Cu^2+^ centers act as electron acceptors for the photoinduced electrons.

### Photocatalytic H_2_ Evolution

Figure 5 illustrates the results of photocatalytic H_2_ production from H_2_O for pristine NaTaO_3_ (NT) and Cu@NaTaO_3_ (NTC) photocatalysts. It is important to underline that these experiments were conducted *without* adding any noble-metal cocatalyst. In Figure 5B, the full H_2_-production kinetic data are presented. Subsequently, Figure 5D presents the rate of H_2_/hour per gram of catalyst; see also Table 3. Across all tested samples, the average experimental standard deviation in H_2_ evolution rates was approximately ±51.4 μmol/g/h, indicating consistent reproducibility of the photocatalytic measurements. The data show that the pristine NT exhibits a marginal photocatalytic H_2_-production performance, i.e., as expected for NaTaO_3_, as well as for all metal-oxide semiconductors, due to the absence of a cocatalyst.^51,74^ For control, NaTaO_3_ plus Cu^2+^ added, i.e., as Cu­(NO_3_)_2_, in the photocatalytic mixture shows insignificant H_2_ production (not shown).

**Figure 5:**
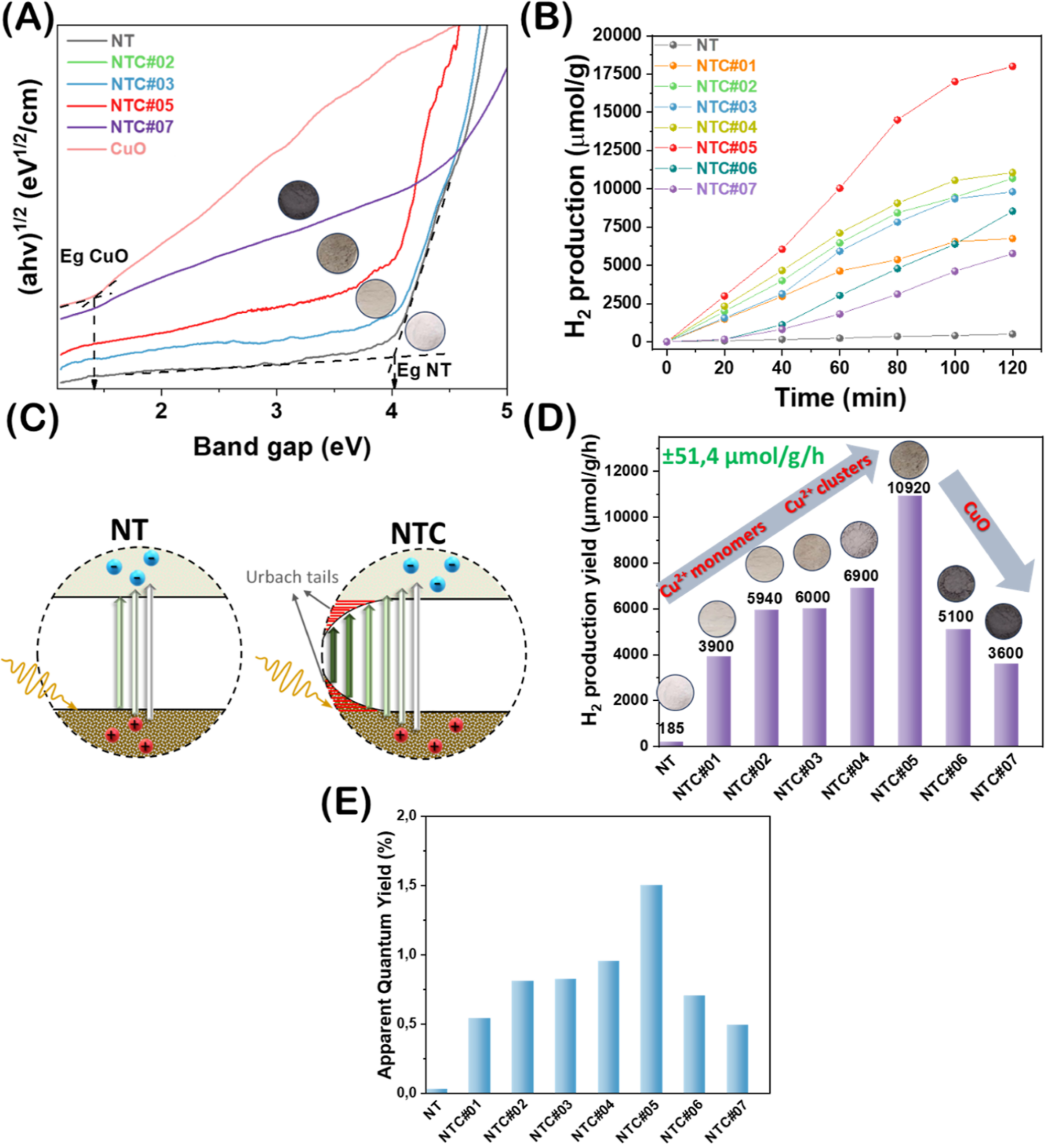
(A) Tauc plots derived from UV–vis DRS spectra to evaluate the band gap energy values of the produced semiconductors, (B) kinetics of photocatalytic H_2_ production of NTC and NT materials under Hg irradiation, (C) schematic illustration of the pristine NT semiconductor without Urbach tails and NTC semiconductors with Urbach tails caused by Cu deposition, (D) H_2_ production yields (μmol/g/h) of NTC and NT materials under Hg irradiation, and (E) apparent quantum yields (%) for H_2_ production.

**Table 3:** Photocatalytic H_2_ Production Rates and Apparent Quantum Yields by X-NaTaO_3_ Catalysts, Where X = Cocatalyst Element

material	H_2_ production rate (μmol/g/h)	apparent quantum yield AQY (%)	cocatalyst % configuration (atom, cluster, particle)	light source	reaction solution	reference
NT	185	0.03	none	Hg (250 W)	H_2_O + 20% methanol	this work
NTC#01	3900	0.54	0.05% Cu (77% monomers + 23% clusters)	Hg (250 W)	H_2_O + 20% methanol	this work
NTC#02	5940	0.81	0.1% Cu (36% monomers + 64% clusters)	Hg (250 W)	H_2_O + 20% methanol	this work
NTC#03	6000	0.82	0.5% Cu (13% monomers + 87% clusters)	Hg (250 W)	H_2_O + 20% methanol	this work
NTC#04	6900	0.95	1% Cu (9% monomers + 91% clusters)	Hg (250 W)	H_2_O + 20% methanol	this work
NTC#05	10,920	1.50	2.5% Cu (6% monomers + 94% clusters)	Hg (250 W)	H_2_O + 20% methanol	this work
NTC#06	5100	0.70	5% Cu (pronominally clusters + CuO NPs ∼3 nm)	Hg (250 W)	H_2_O + 20% methanol	this work
NTC#07	3600	0.49	10% Cu (pronominally clusters + CuO NPs ∼3 nm)	Hg (250 W)	H_2_O + 20% methanol	this work
1% Pt–NaTaO_3_	350/6100	0.84	1% Pt photodeposition (1–2 nm Pt NPs)	Xe (250 W)/Hg(250 W)	H_2_O + 20% methanol	this work
1% Pt–NaTaO_3_	350		1% Pt photodeposition (1–2 nm Pt NPs)	Xe (250 W)	H_2_O + 20% methanol	^10^
NiO/NaTaO_3_	391		0.5% NiO (2 nm NiO NPs)	Xe (250 W)	H_2_O + 20% methanol	^10^
NiO/NaTaO_3_	2.180		0.05% NiO	Hg (400 W)	H_2_O	^75^

In contrast, in Cu@NaTaO_3_, the deposition of Cu^2+^ on NaTaO_3_ via FSP noticeably enhanced the photocatalytic performance of NaTaO_3_, see Figure 5B,D. Specifically, materials NTC#01 and NTC#02, with low Cu^2+^ content, according to EPR—mainly Cu^2+^ monomers on the particle’s surface—exhibited H_2_ production of 3900 and 5940 μmol/g/h, respectively, see Figure 5D and Table 3.

These H_2_-production rates correspond to apparent quantum yields^76^ of 0.54 and 0.81%, see calculation details in Supporting Information, Figure 5E, and Table 3. Most intriguingly, materials NTC#03, NTC#04, and NTC#05, i.e., with increasing Cu^2+^ content/with—according to EPR—predominant Cu^2+^ clusters, show a considerable boosting of H_2_ production, reaching a maximum yield of 10,920 μmol/g/h (AQY = 1.5%) in the case of NTC#05. To our knowledge, this is the highest H_2_ production efficiency reported so far for NaTaO_3_ with a non-noble-metal cocatalyst. For comparison, our 1% Pt/NaTaO_3_ archives production of 6100 μmol/g/h (AQY = 0.84%) (Figure S4 in Supporting Information); thus, NTC#05 is a far superior catalyst than 1% Pt/NaTaO_3_. For completeness, we notice that the 1% Cu/NaTaO_3_ sample (NTC#04) exhibits a comparable H_2_ production rate (AQY = 0.95%) and even surpasses the reference 1% Pt/NaTaO_3_ (AQY = 0.84%). At higher Cu concentrations, NTC#06 and NTC#07, where, according to Raman and XRD, CuO formation is favored, the H_2_ production yield rapidly decreases at 5100 and 3600 μmol/g/h for NTC#06 and NTC#07, respectively. The data in Figure 5E clearly demonstrate that the enhanced photoactivity boosted by the Cu nanoclusters originates from a boosting of the apparent quantum yields. This implies that the beneficial role of the Cu nanoclusters is linked to the fundamental steps of photoexcited electron dynamics in our Cu–NaTaO_3_. Hereafter, we provide direct evidence using EPR spectroscopy that the Cu nanoclusters are the optimal shuttles for photogenerated electrons from NaTaO_3_ toward H_2_. In the same context, the TOF values were calculated by dividing the hydrogen evolution rates by the molar amount of Cu in monomeric and clustered configurations, as quantified by EPR spectroscopy (Table S1). The results reveal that TOF per Cu monomer is highest at very low Cu loadings, reflecting their isolated, under-coordinated environment. However, this activity is only maintained within a narrow loading range, and the total number of available monomeric sites remains low. In contrast, Cu subnanoclusters show slightly lower TOF per site, particularly at higher loadings where some aggregation may occur, but their greater abundance leads to a much higher overall H_2_ production rate and AQE. Importantly, samples such as NTC#05, which are dominated by subnanometric Cu clusters, exhibit the highest hydrogen evolution rates and apparent quantum yields (AQE = 1.5%), suggesting that under the conditions studied, Cu subnanoclusters are more effective than monomers in driving high H_2_ evolution rates, especially when combined with increased cocatalyst loading. These results strongly support our conclusion that subnanoclusters provide an optimal balance between site activity, charge separation efficiency, and population density.

Overall, the present photocatalytic data demonstrate that [i] Cu subclusters (Cu/SnC), *not* Cu^2+^ single atoms *or* small CuO particles, are the optimal cocatalysts for NaTaO_3_ toward photocatalytic H_2_ production. [ii] The Cu­(SnC)/NaTaO_3_ catalyst is a highly efficient H_2_O/H_2_-photocatalyst able to operate with no nobble metal as a cocatalyst. The optimal NTC#05 Cu­(SnC)/NaTaO_3_ achieves an H_2_ production far superior to reference 1% Pt/NaTaO_3_.

For a better understanding of the interfacial dynamics of the cocatalytic Cu moieties on NaTaO_3_, UV–vis diffuse reflectance spectroscopy (DRS) was employed to determine the bandgap energy of the NTC nanoparticles, applying the Tauc plot, using the Kubelka–Munk method, according to eq 6:

6 $$ahv=K{\left(hv-E_{g}\right)}^{p}$$

where α = absorption coefficient, *hv* = photon energy, *E*_g_ = bandgap, *K* = constant, and *p* = 1/2 for direct-band transition.^77^ We underline that, as discussed previously,^78^ to avoid mistakes, the analysis of the Kubelka–Munk plots to estimate the band gap should be done carefully, i.e., taking into account the correct baseline, *not* simply the intersection of the tangent with axis-X,^79^ see dotted lines in Figure 5A.

The data in Figure 5A show that, upon increase of Cu loading, a gradual rise in absorbance of visible-light photons at <3 eV, i.e., visually anticipated by the color change of the powders (see inset photos in Figure 5A): the crispy-white NT attains a dark-gray tint at high-Cu-loading NTC#01-to-7. Based on the data, the bandgap energies of all the semiconductors are listed in Table 1. For NT, NTC#01–05 in cases, the bandgap values, when correctly estimated,^78^ are approximately at *E*_g_ = 4 eV, which is consistent with the reported values for NaTaO_3_ nanostructures in the literature.^80,81^ This shows that the deposited Cu^2+^ ions exert little effect on the bandgap of the perovskite since they are *not* embedded *into* the NaTaO_3_ lattice but only at the surface.

In this context, it is pertinent to clarify that the observed tails in NTC#01–05 are attributed to the formation of the so-called Urbach tails,^78,82^ i.e., small distortions of the density of states profiles at the low edge of the conduction band and the upper edge of the valence of NaTaO_3_.^18,83^ These tails are typically of the order of <100 meV due to the slight smearing-out of the Kubelka–Munk plot near the band gap energy (see also scheme in Figure 5C). We underline that an analogous phenomenon is expected in the case of lattice vacancies;^18^ however, in the present case, we exclude this since EPR and Raman definitely show no vacancies in the Cu@NaTaO_3_ materials, i.e., as expected from the oxygen-rich combustion FSP process used to produce the NaTaO_3_. Taking into account the AQY data in Figure 5E and Table 3, we consider that the reorganization of the DOS of NaTaO_3_ via the Urbach tails is one of the mechanisms that contribute to the beneficial role of the Cu nanoclusters on the H_2_ photogeneration. In addition, our EPR data show that the Cu nanoclusters are the optimal shuttles for photogenerated electrons from NaTaO_3_ toward H_2_.

Finally, for the high-Cu-loading materials (NTC#06, NTC#07), a second bandgap emerges, corresponding to the *E*_g_ of the small CuO particles, at approximately 1.5 eV.^84,85^ This observation is in accordance with XRD, EPR, and Raman spectroscopy, which show the formation of small CuO particles of copper oxide in NTC#06 and NTC#07.

## Discussion

### On the Dynamics of the Cu Moieties on the NaTaO_3_

The preset data provide a wealth of novel information on the interfacial Cu–NaTaO_3_ association and its relation to the remarkable boosting of photocatalytic H_2_ production, optimally by the Cu subnanoclusters in NTC#05: the present data clearly show that the association of the Cu with the NaTaO_3_ *surface*, not the interior of the lattice, is the key phenomenon. In this context, to further peer into the dynamics of the Cu cocatalyst on the surface of NaTaO_3_ nanoparticles, thermal (750 °C, *t* = 120 min) post-FSP treatment experiments were conducted and monitored in situ by Raman spectroscopy. The key hypothesis was that this post-FSP thermal treatment could alter the Cu-moieties arrangement, for example, toward the formation of clusters/particles. The Raman spectra, in Figure 6A, confirm this: post-FSP treatment materials (NTC-PT) increase the aggregation state of the Cu cocatalyst toward clusters and eventually to CuO small nanoparticles, which results in a surface distortion of NaTaO_3_, compared to the as-prepared (NTC). This distortion is detectable clearly by Raman spectroscopy since the deformation bonds of O–2Ta or O–3Ta are mostly affected, as shown in Figure 3B. Specifically, the Raman spectra of NTC#04, which is a mixture of Cu^2+^ monomers and Cu^2+^ clusters, upon thermal treatment (NTC-PT) tend to be similar to those of NTC#05, which contains a higher Cu^2+^ cluster population. In the same context, after the thermal treatment of NTC#05, the formation of CuO nanoparticles is easily detected by the characteristic bands at 210 and 610 cm^–1^ (Figure 6A) as well as in XRD (Figure 6C).

**Figure 6:**
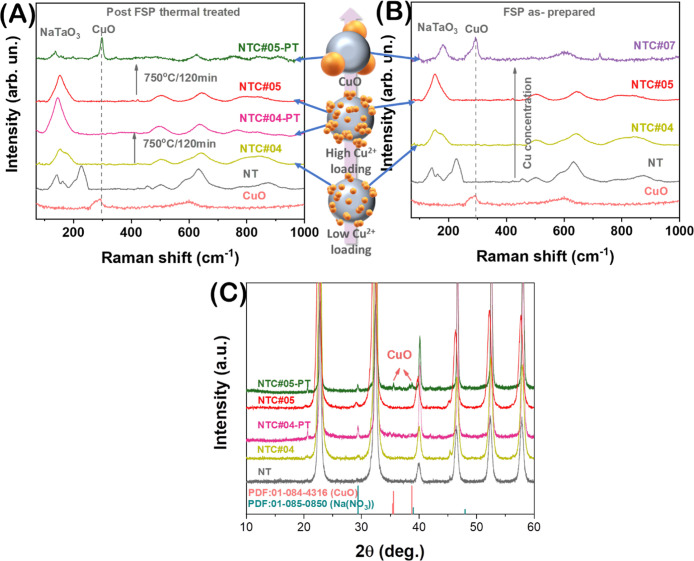
(A) Raman spectra of the NTC samples after post-FSP heat treatments at 750 °C for 120 min, (B) Raman spectra of the as-prepared NTC samples, and (C) XRD patterns of NTC-PT samples compared to the pristine ones.

The above data allow a better understanding of the disposition/dynamics of the Cu moieties on the NTC materials: upon heating at high *T* (750 °C), while the Cu^2+^ concentration remains stable, the aggregation state of the Cu^2+^ cocatalyst increases. The as-prepared FSP-made nanocatalysts are initially decorated with highly dispersed Cu^2+^ monomers and/or Cu^2+^ clusters, depending on the selected Cu^2+^ concentration. Following post-FSP heat treatment, the Cu^2+^ monomers migrate across the semiconductor surface and aggregate to form Cu^2+^ clusters. Sequentially, Cu^2+^ clusters further aggregate to form small CuO nanoparticles.

### Monitoring the Photoinduced Electron Transfer from NaTaO_3_ to Cu Cocatalysts by In Situ EPR

The transfer of electrons to Cu^2+^ results in their reduction to Cu^1+^ (Cu^2+^ + e^–^) and eventually to Cu^0^ (Cu^1+^ + e^–^ or Cu^2+^ + 2e^–^). Since Cu^1+^ and Cu^0^ are EPR silent, monitoring of the Cu^2+^ population allows quantitative monitoring of the e^–^-transfer kinetics when NaTaO_3_ is photoexcited in NTC materials. Here, to ensure maximum e^–^-transfer efficiency, we used methanol as a typical hole scavenger in photocatalysis. In this way, we monitored the kinetics of the Cu^2+^-EPR spectra behavior of NTC#02 and NTC#05 under 275 nm irradiation, as shown in Figure 7.

**Figure 7:**
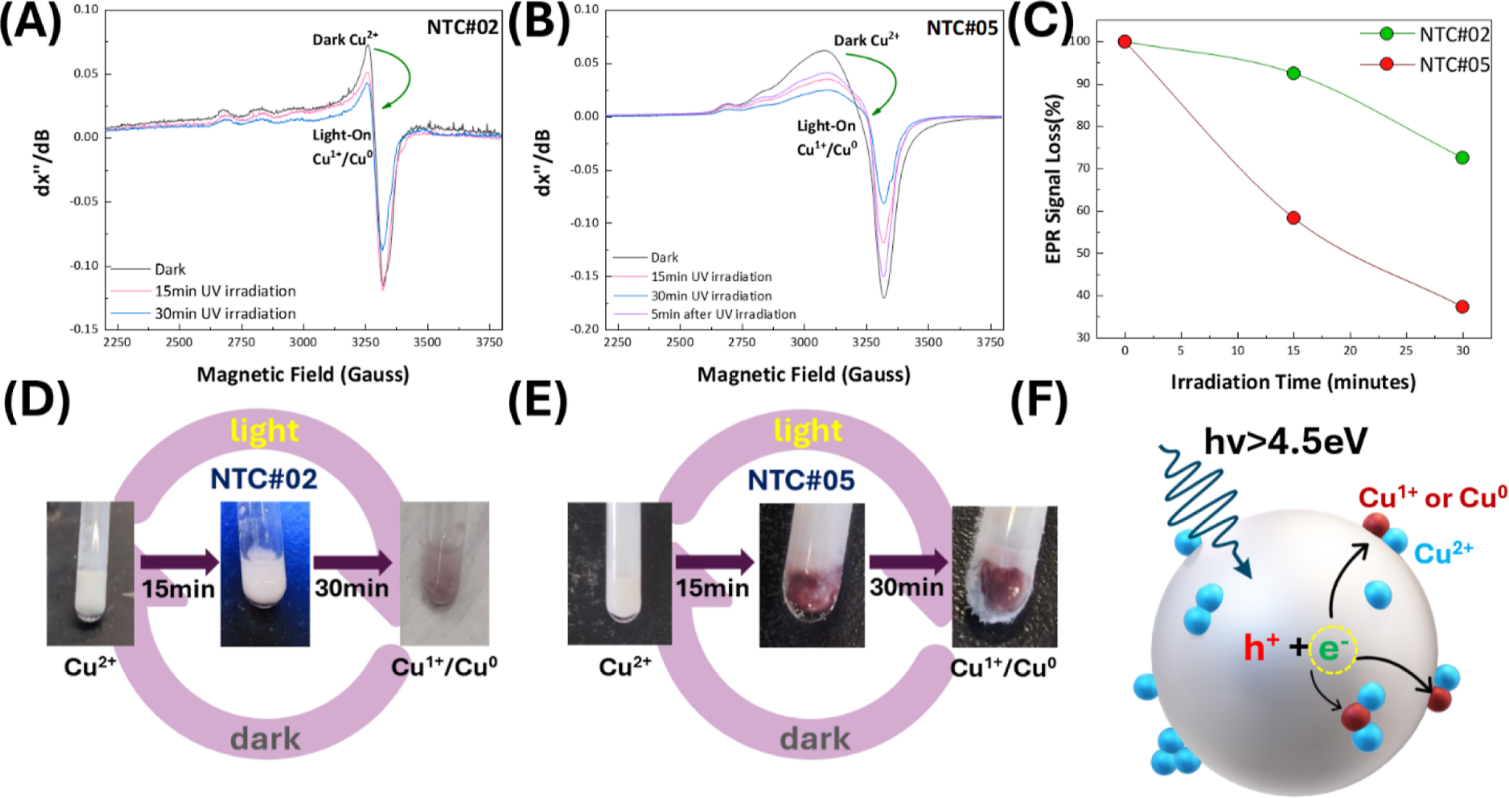
EPR spectra recorded at 77 K in the presence of methanol, acting as a hole scavenger, under illumination for materials (A) NTC#02 and (B) NTC#05. (C) Quantified EPR signals corresponding to the spectra of figures (A) and (B). Upon prolonged irradiation (*hv* > 4.5 eV), the Cu^2+^ signals decrease, indicating the conversion of Cu^2+^ ions into either Cu^1+^ or Cu^0^, both of which are EPR-silent. (D,E) Photographs of NTC#02 and NTC#05 during UV irradiation, showing a color change in both materials. This change is more pronounced for NTC#05, correlating with a greater loss of EPR signal. (F) Schematic representation of NTC perovskites under UV irradiation where photogenerated electrons migrate on the surface of the semiconductor and reduce the Cu^2+^ species that are present there.

The Cu^2+^-EPR spectra of irradiated NTC#02 and NTC#05 (Figure 7A–C) display a similar trend, with a progressive Cu^2+^-EPR signal loss, which verifies the reduction of Cu^2+^ ions to either Cu^1+^ or Cu^0^, both of which are EPR-silent. A closer inspection of the EPR spectra for the photocatalyst under illumination is presented in Figure S6 in the Supporting Information. Specifically, we observe that in the best-performing photocatalyst NTC#05, under illumination, a *g* = 2.052 signal is developed. The photoinduced signal is reversible, i.e., after switching off the light, this signal rapidly disappears in the dark, as shown in Figure S6A. This provides strong evidence that it originates from a light-induced electron-transfer process. In the low-Cu-loading material NTC#02, this EPR is much lower, as shown in Figure S6B; thus, we conclude that it originated from a reversible e^–^ transfer to Cu^2+^. The line shape and the *g* = 2.052 value are compatible with the EPR signals of small Cu(0) clusters or ultrasmall particles.^86^ For completeness, we underline that in such systems, the EPR signal corresponds to an effective *S* = 1/2 spin state; however, it does not originate from any steady-state unpaired electron in the Cu(0) system. As analyzed originally by Feher and Kip^87^ and verified by Tikhonov et al.^86^ for small Cu(0) particles and by us^88^ in the case of small Ag nanoparticles, the nonzero spin results from an effective spin imbalance due to the collective thermodynamics of the spin in the small metal particle. The exact EPR line shape depends on the size of the small metal structure as well as the measuring temperature. Regarding the photocatalytic mechanism, overall, the present in situ EPR data show that in Cu/NaTaO_3_ under light, photoinduced electron transfer from NaTaO_3_ to the surface Cu^2+^ species results in reduction of Cu^2+^ that forms paramagnetic Cu(0) (4s^1^3d^10^) (*S* = 1/2) species. The intermediate Cu^1+^ (3d^10^) (*S* = 0) state is EPR-silent.

The Cu-reduction process indicates the photoinduced electron transfer from the NaTaO_3_ matrix to surface Cu^2+^ ions, evidenced also by the noticeable color change that occurs, as depicted in Figure 7D,E, i.e., the pink-red color is indicative for Cu^1+^and/or Cu^0^.^89,90^ Notably, the kinetics of the Cu reduction, depicted in Figure 7, is much faster in NTC#05 than in NTC#02. Especially, according to Figure 7E, during the initial 15 min of irradiation, NTC#05 exhibits >40% reduction of the Cu^2+^ centers vs 7% observed for NTC#02. At prolonged irradiation, the color of NTC#02 shifts to a pale pink, whereas NTC#05 develops a deep burgundy hue, correlating with an approximately 60% Cu^2+^ signal loss in NTC#05 compared to ∼25% in NTC#02, as shown in Figure 7E. This color change can be visualized in the schematic representation of Figure 7F: UV photons excite NaTaO_3_ hole–electron pairs, where the holes are scavenged through the oxidation of methanol, while electrons migrate to the surface of the semiconductor in order to participate in surface reactions like H_2_ production. Consequently, the Cu-EPR kinetics provide further direct evidence that Cu subnanoclusters serve as more efficient e^–^ acceptors than Cu monomers, confirming the mechanism of enhanced photocatalytic H_2_ production by the Cu subnanoclusters in NTC#05 and NTC#06 and particularly NTC#07. Overall, the present data indicate that the beneficial effect of the Cu nanoclusters arises from their ability to enhance the apparent quantum yield by improving photon utilization, induce reorganization of the density of states in NaTaO_3_ through Urbach tail formation, and efficiently mediate the transfer of photogenerated electrons from NaTaO_3_ to hydrogen.

### Comments on the Cocatalytic Role of Cu Subnanoclusters vs Single Cu Atoms

The present data demonstrate that Cu subnanoclusters, *not* single Cu atoms, are the optimal Cu configuration as cocatalysts for NaTaO_3_. As we discussed in our recent review article,^40^ this finding entails that other cases of Cu-loaded semiconductors might need to be considered with diligence, i.e., to clarify the difference between Cu-ScN vs CuSAC. For example, it is pertinent to notice that in ref 47, the authors stated that single-atom catalysts are their optimal cocatalysts; however, their EPR data, Figure 4 in ref 47, clearly indicates that they correspond to Cu clusters, similar to the present NTC#03 (see Figure S5). This is of importance since the quantum mechanical properties and the ensuing photo/redox physics of Cu clusters are fundamentally different from isolated Cu atoms. Cu clusters tend to resemble more quantum dots with semidiscrete energy levels. In this context, the present work provides targeted clarification of this issue.

## Conclusions

Cu/NaTaO_3_ nanocatalysts were synthesized using an innovative flame spray pyrolysis (FSP) process, which enabled the creation of a diverse library of perovskite nanoparticles decorated with varying Cu^2+^ concentrations in a single step, producing highly crystalline nanocatalysts with integrated cocatalysts at different aggregation states. The presence of Cu on the NaTaO_3_ surface was confirmed through XPS, XRD, and HRTEM analyses, while its precise configuration and quantification were determined by using EPR spectroscopy. HRTEM images and Raman spectroscopy further revealed that as Cu^2+^ aggregates, the catalyst’s surface undergoes distortion, enhancing metal–support interactions between the catalyst and cocatalyst. Photocatalytic H_2_ production experiments show that NaTaO_3_ decorated with Cu^2+^ subnanoclusters achieves significantly higher activity than its single Cu-atom counterpart, achieving 10.920 μmol H_2_/g/h. In situ EPR elucidated the photoinduced electron transfer from NaTaO_3_ to surface-anchored Cu^2+^, with Cu^2+^ subnanoclusters exhibiting approximately three times higher efficiency than Cu^2+^ monomers. The beneficial action of the Cu nanoclusters can be attributed to [i] improved apparent quantum yield, i.e., more efficient overall utilization of the photons, and [ii] the reorganization of the DOS of NaTaO_3_ via the Urbach tails. [iii] Cu nanoclusters are the optimal shuttles for photogenerated electrons from NaTaO_3_ toward the H_2_.

## Supplementary Material



## Notes

Experimental details; additional XRD; Tauc plots derived from UV–vis DRS spectra; photocatalytic H_2_ production kinetics and production yields of 1% Pt/NaTaO_3_ and 1% Cu/NaTaO_3_; EPR spectra recorded at 77 K of materials NTC#01, #03, #05, and #06; light-induced EPR spectra focusing on the *g* ∼ 2 region of NTC#05 and NTC#02 photocatalysts; TOF and AQE calculation methods; TOF data; and additional photocatalytic results (PDF)
