# Supplementary material for: Atomic Cu2+ Clusters vs Single Cu2+ Atoms as Optimal Cocatalysts on NaTaO3 for Enhanced Noble-Metal-Free H2O/H2 Photocatalysis
Source: ACS Catal. 2025 Jul 23;15(15):13595–610. doi: 10.1021/acscatal.5c03183 (PMC12323387; doi:10.1021/acscatal.5c03183)
Supplement: Supplementary file 1 [file cs5c03183_si_001.pdf]

Supporting Information

Atomic Cu^{2+}-clusters *vs.* Single-Cu^{2+}-Atoms as optimal co-catalyst on $NaTaO_3$ for enhanced noble-metal-free H_2O/H_2-photocatalysis.

*Anastasia V. Spyrou, Areti Zindrou, Christos Sidiropoulos, Yiannis Deligiannakis**

Laboratory of Physical Chemistry of Materials & Environment, Department of Physics, University of Ioannina, Ioannina, Greece

* Corresponding author: Yiannis Deligiannakis (ideligia@uoi.gr)

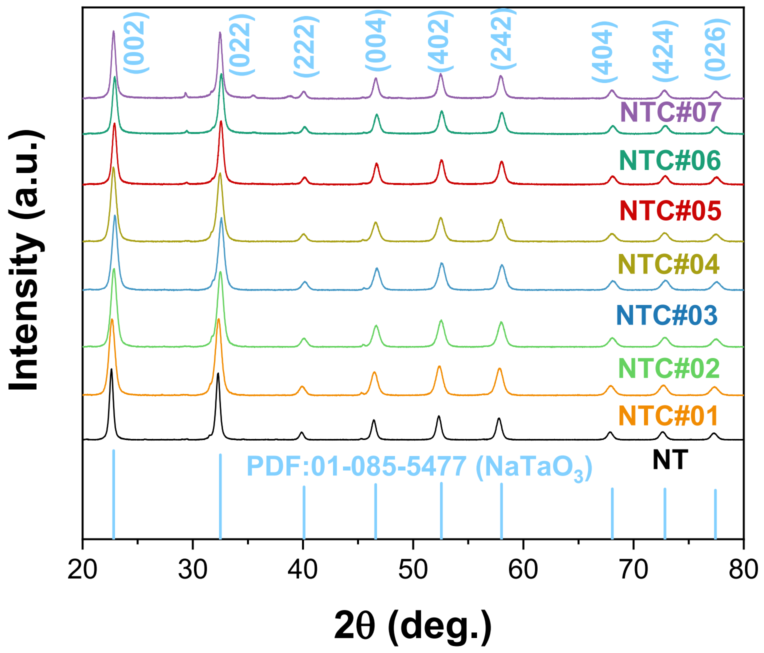

Figure S1. XRD patterns of the NTC samples and pristine $NaTaO_3$.

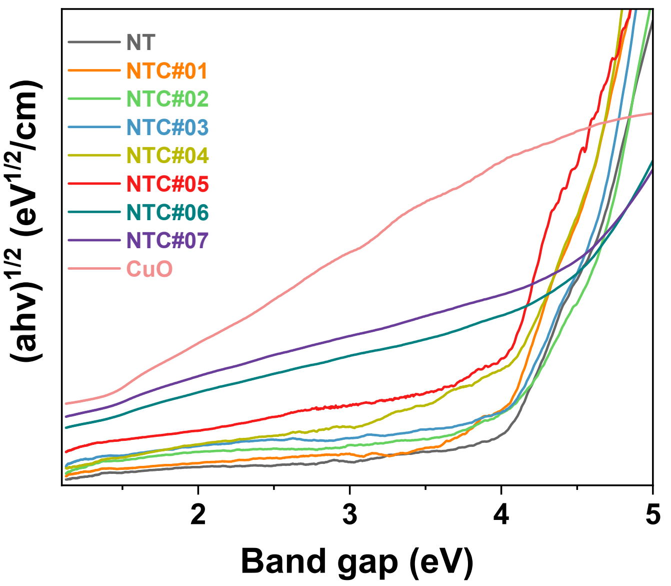

Figure S2. Tauc plots derived from UV−Vis DRS spectra to evaluate the band gap energy values of the produced NTC samples compared to the pristine $NaTaO_3$ and CuO.

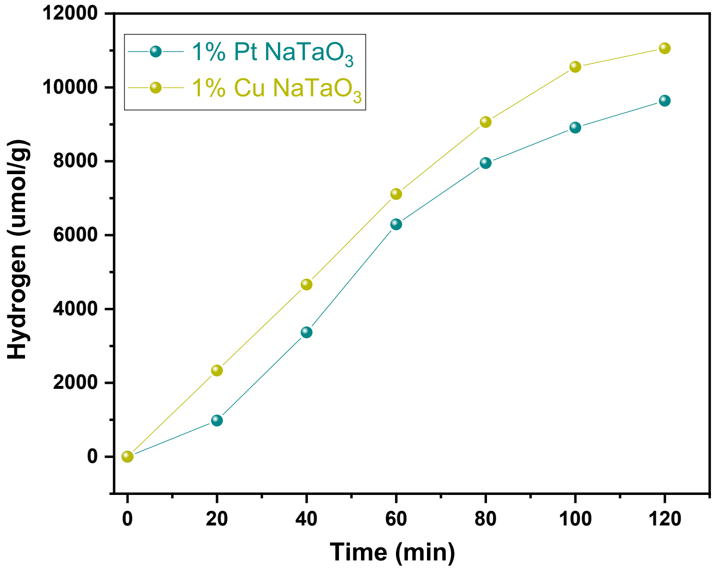

Figure S3. Kinetics of Photocatalytic H_2 production of 1% Pt/$NaTaO_3$ compared to 1% Cu/$NaTaO_3$ materials under Hg irradiation.

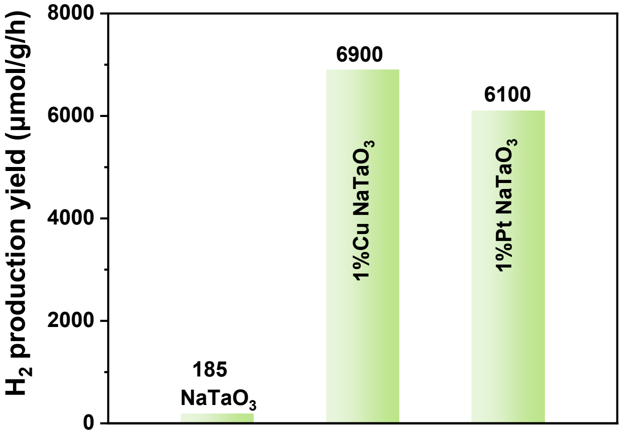

Figure S4. H_2 production yields (µmol/g/h) of 1% Pt/$NaTaO_3$ compared to 1% Cu/$NaTaO_3$ and $NaTaO_3$ under Hg irradiation.

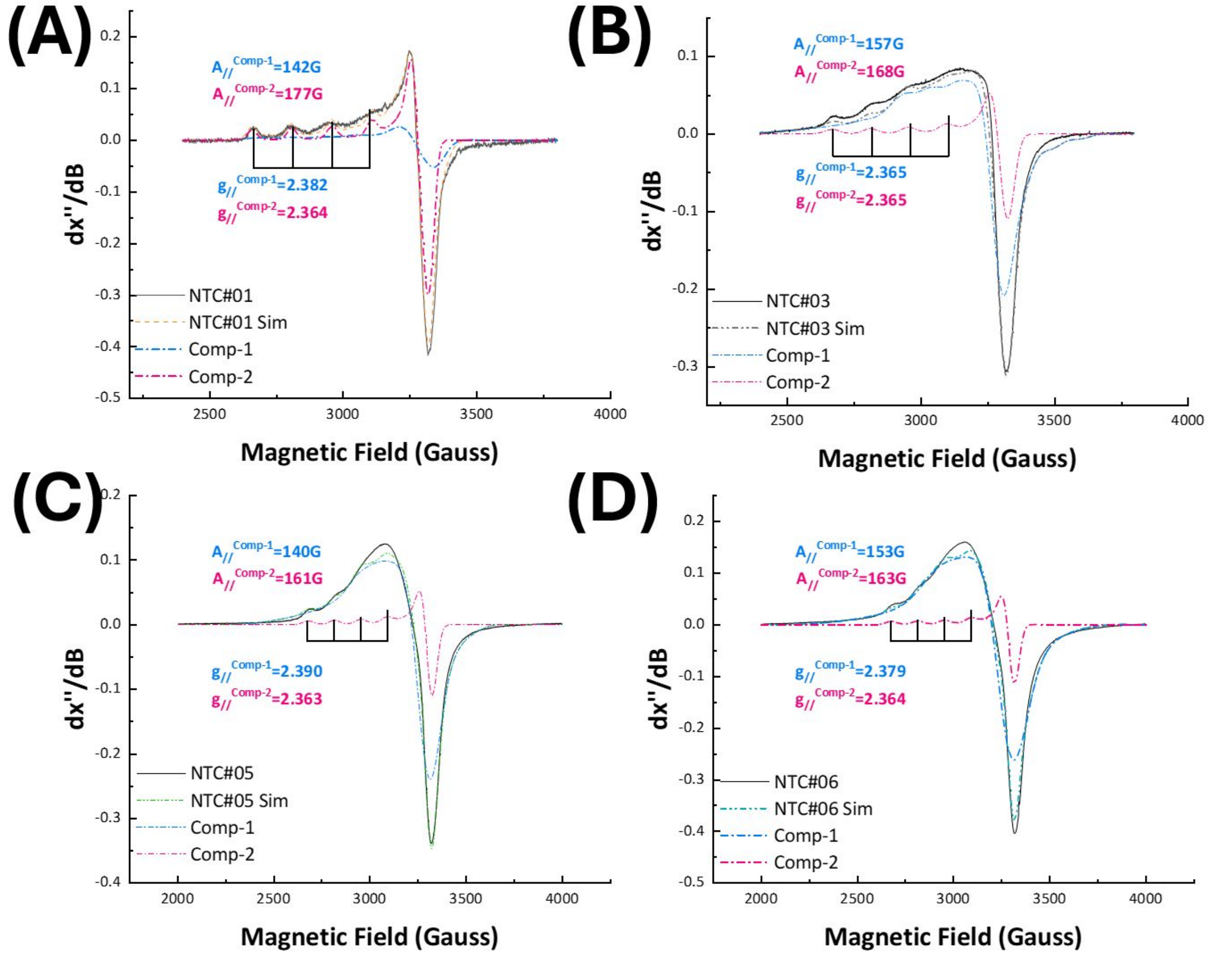

Figure S5. EPR spectra recorded at 77K of materials NTC#01, NTC#03, NTC#05 and NTC#06. Solid lines represent the experimental spectra while the dotted lines represent the simulated EPR spectra

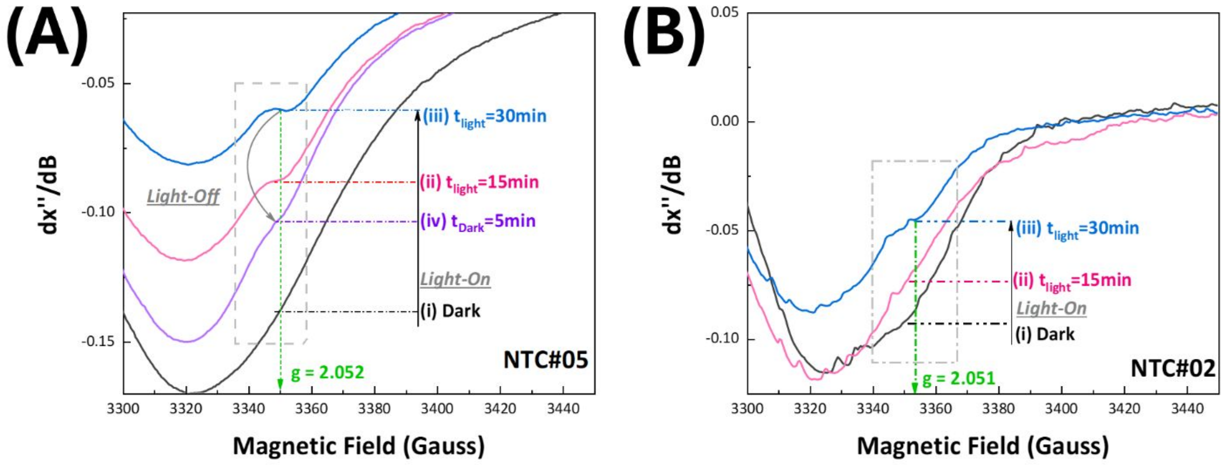

Figure S6 Light-Induced changes in the EPR spectra, (same EPR spectra as in Figure 7) focused on the g~2 region. (A) NTC#05 photocatalyst, (B) NTC#02 photocatalyst. In (A) i.e. $NaTaO_3$ with Cu-nanoclusters, under light, the g=2.052 Cu^0(S=1/2, $4s^13d^{10}$) signal, see dashed rectangular frame, is formed at the expense of the main Cu^{2+}(S=1/2)-EPR signal that diminishes i.e. due to photoreduction of to Cu^{2+}(S=1/2) to Cu^{1+} (S=0) and Cu^0 (S=1/2, $4s^13d^{10}$). This Cu^0 is maximized after 30 minutes of irradiation, spectrum (A)(iii-blue). After switch-off the light the EPR signal is reversibly disappearing i.e. due to the back-oxidation of $Cu^{(0)}$ to Cu^{2+}. In (B), monomeric Cu-atoms on $NaTaO_3$, the electron-transfer to Cu^{2+} is less efficient i.e. the Cu^{2+} does not change by much, and the $Cu^{(0)}$ is formed to a lesser extent.

Apparent Quantum Yield (AQY%) Calculation

Apparent Quantum Yield (AQY)%[1], was calculated using the following equations[2][3]:

$$AQY(\%) = \frac{2 \times N_{H2}}{2\text{Nphotons}} \times 100\ \% \quad \text{(S1)}$$

where N_{H2} is the number of H_2 molecules and Nphotons is the number of incident photons:

$$Nphotons\ = \frac{E\lambda}{hc} = \frac{ISt\lambda}{hc} \quad \text{(S2)}$$

where I= Light intensity (I_{252nm} =140 mW cm^{-2}), S = Irradiation area (94 cm^2), t= Irradiation time (h), λ = 252 nm, h = Planck constant, c =speed of light.

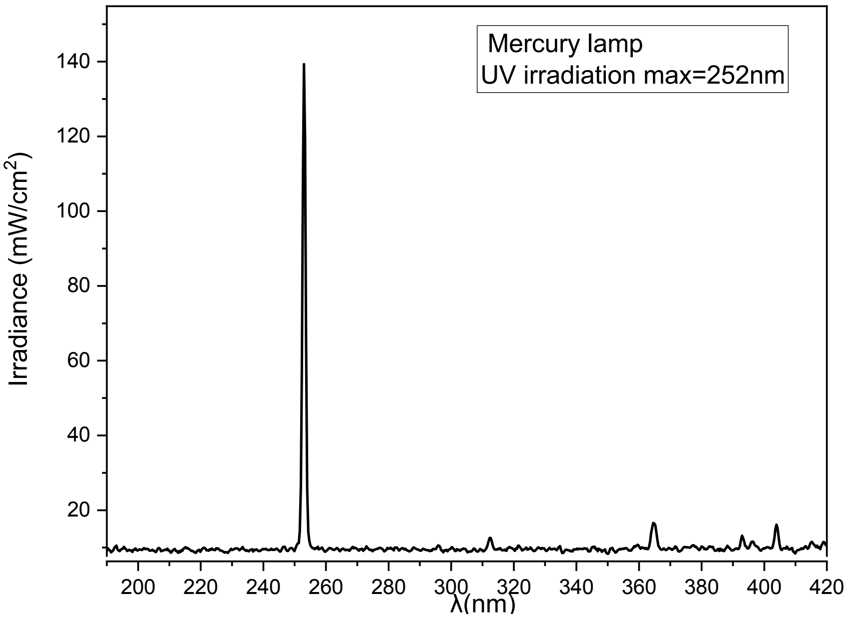

Figure S7. Power emission spectrum of our mercury lamp, (mW/cm^2) as a function of wavelength.

Based on the irradiation power at 252 nm, the incident photons were calculated using relation S2 to be $Nphotons = 6.39 \times 10^{21}$. Accordingly, the AQY% calculated using equation S1 are listed in Table 3, in the main text.

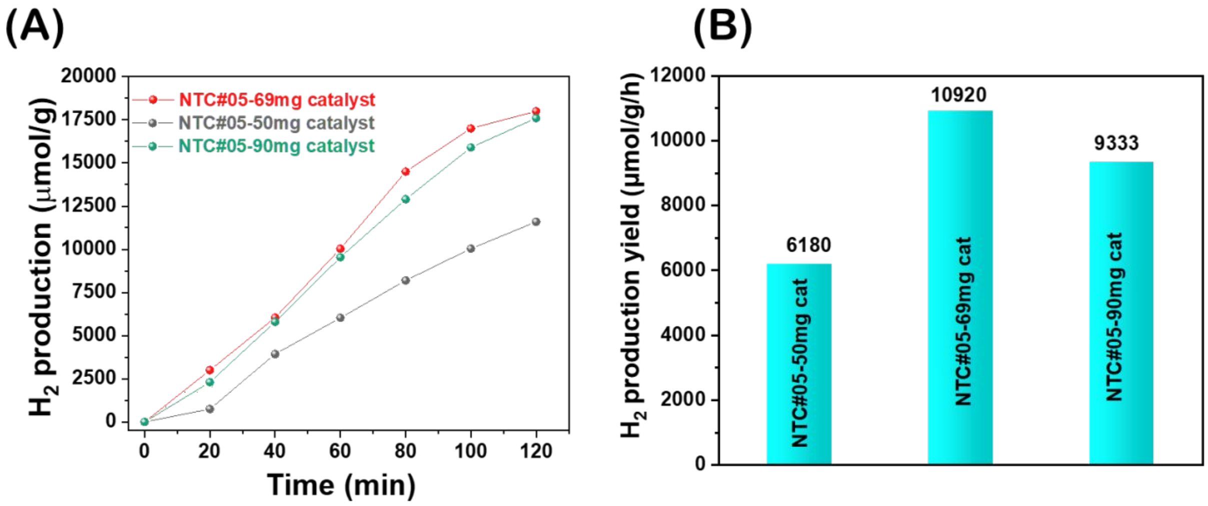

Figure S8. Photocatalytic H_2 production kinetics and yield ($\mu mol\, g^{-1}\, h^{-1}$) of NTC#05 using different catalyst masses (50, 69, and 90 mg) under mercury lamp irradiation.

Calculation of TOF (s^{-1}) values:

$$TOF = \frac{\mathrm{H_2}\ production\ rate\ (\frac{mol}{s})}{\text{mol of active Cu cites}} \quad \text{(S3)}$$

Table S1. Photocatalytic H_2 production rates, TOF (s^{-1}) values, and Apparent Quantum Yields, by Cu-$NaTaO_3$ catalysts

Material	Cu loading (%w/w)	Cu Species(%)	H_2 Rate (μmol/g/h)	TOF (s^{-1})	AQY (%)
**NTC#01**	0.05	Monomers (77)	3900	0.179	0.54
		Clusters (23)		0.599	
**NTC#02**	0.1	Monomers (36)	5940	0.291	0.81
		Clusters (64)		0.164	
**NTC#03**	0.5	Monomers (13)	6000	0.163	0.82
		Clusters (87)		0.024	
**NTC#04**	1	Monomers (9)	6900	0.135	0.95
		Clusters (91)		0.013	
**NTC#05**	2.5	Monomers (6)	10920	0.129	1.50
		Clusters (94)		0.0082	
**NTC#06**	5	Monomers (3)	5100	0.060	0.70
		Clusters (97)		0.00186	
**NTC#07**	10	Monomers (0)	3600	0	0.49
		Clusters (100)		0.00064	

References

[1] M. Melchionna and P. Fornasiero, "Updates on the Roadmap for Photocatalysis," *ACS Catal.*, vol. 10, no. 10, pp. 5493–5501, May 2020, doi: 10.1021/acscatal.0c01204.

[2] C. Acar, I. Dincer, and G. F. Naterer, "Review of photocatalytic water-splitting methods for sustainable hydrogen production," *Int. J. Energy Res.*, vol. 40, no. 11, pp. 1449–1473, 2016, doi: https://doi.org/10.1002/er.3549.

[3] A. Zindrou, L. Belles, M. Solakidou, N. Boukos, and Y. Deligiannakis, "Non-graphitized carbon/Cu2O/Cu0 nanohybrids with improved stability and enhanced photocatalytic H2 production," *Sci. Rep.*, vol. 13, no. 1, p. 13999, 2023, doi: 10.1038/s41598-023-41211-4.
